# Exosome-based delivery strategies for tumor therapy: an update on modification, loading, and clinical application

**DOI:** 10.1186/s12951-024-02298-7

**Published:** 2024-01-28

**Authors:** Qian Yang, Shisheng Li, Haibo Ou, Yuming Zhang, Gangcai Zhu, Shaohong Li, Lanjie Lei

**Affiliations:** 1 Department of Otorhinolaryngology Head and Neck Surgery, The Second Xiangya Hospital, Central South University, Changsha, 410011 Hunan China; 2 Institute of Translational Medicine, Zhejiang Shuren University, Hangzhou, 310015 Zhejiang China

**Keywords:** Exosome, Isolation, Surface functionalization, Drug delivery, Tumor therapy

## Abstract

Malignancy is a major public health problem and among the leading lethal diseases worldwide. Although the current tumor treatment methods have therapeutic effect to a certain extent, they still have some shortcomings such as poor water solubility, short half-life, local and systemic toxicity. Therefore, how to deliver therapeutic agent so as to realize safe and effective anti-tumor therapy become a problem urgently to be solved in this field. As a medium of information exchange and material transport between cells, exosomes are considered to be a promising drug delivery carrier due to their nano-size, good biocompatibility, natural targeting, and easy modification. In this review, we summarize recent advances in the isolation, identification, drug loading, and modification of exosomes as drug carriers for tumor therapy alongside their application in tumor therapy. Basic knowledge of exosomes, such as their biogenesis, sources, and characterization methods, is also introduced herein. In addition, challenges related to the use of exosomes as drug delivery vehicles are discussed, along with future trends. This review provides a scientific basis for the application of exosome delivery systems in oncological therapy.

**Graphical Abstract:**

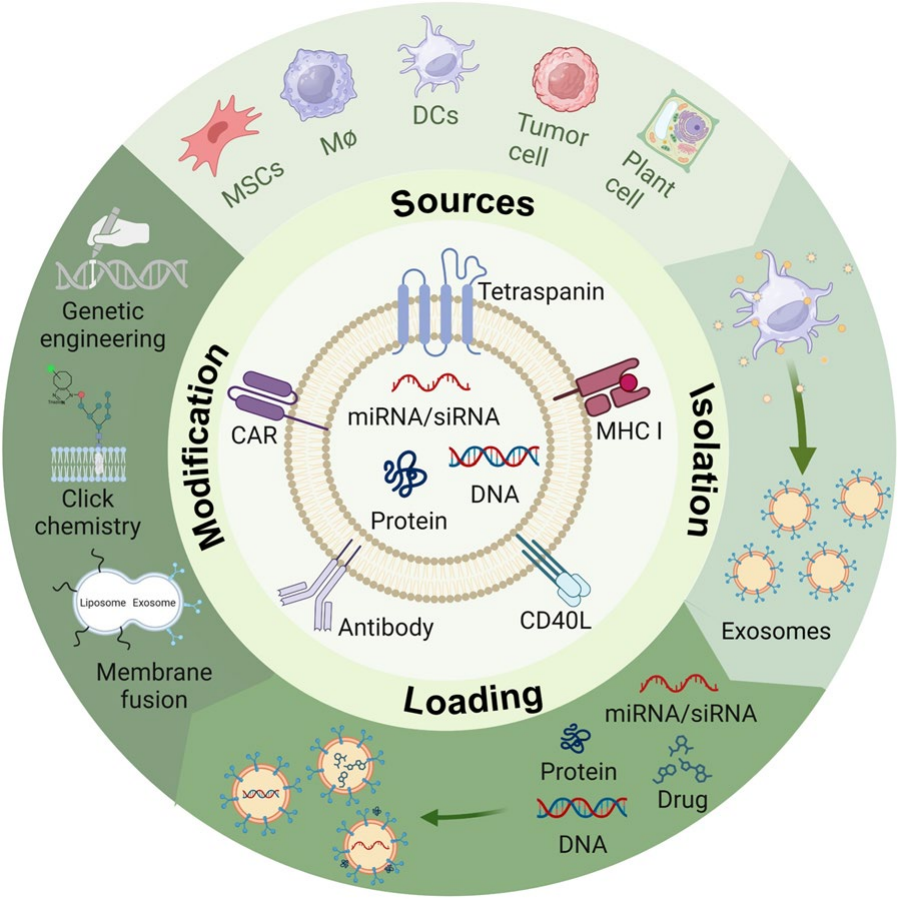

## Introduction

Malignancy is a major public health problem and among the leading lethal diseases worldwide. With the continuous development of preventive measures in recent years, alongside early diagnosis and treatment methods related to tumors, the mortality rate of malignant tumors has shown a certain degree of reduction [1]. The current primary methods used to treat tumors include surgery, chemotherapy, radiotherapy, immunotherapy, targeted therapy, and optical therapy. Despite the effectiveness of various drugs in tumor treatment, shortcomings such as poor water solubility, short half-life, and local and systemic toxicity remain [2]. Unfortunately, normal cells and tissues usually suffer inevitable damage from therapies targeting tumor cells. Therefore, the delivery of therapeutic agents for achieving safe and efficient antitumor therapy is an urgent challenge in this field.

In recent years, the emergence of liposomes and nanoparticles has offered new possibilities for the delivery of antitumor drugs by allowing additional targeting and controlled release. Liposomes are spherical or spheroidal structures composed of lipid bilayers that encapsulate drugs and protect them from degradation, while also increasing their concentration at the tumor site. Nanoparticles are nanoscale drug carriers with high drug loading and stability capacities that are made of polymers, proteins, or other biocompatible materials. In addition, surface modification of nanoparticles can result in specialized targeting and controlled release of drugs, therefore increasing drug selectivity for tumor tissues while reducing the damage to normal tissues and cells. Compared with free drugs, both liposomes and nanoparticles have shown superior antitumor effects with less side effects both in vitro and in vivo. In addition, due to the high permeability of tumor vasculature and poor lymphatic drainage, nanoparticles can extravasate into the tumor site, promoting the enhanced accumulation of nanoparticles in the tumor [3]. In addition to the delivery of chemotherapeutic agents, the combination of immunotherapy, photodynamic therapy and other therapies can further enhance the efficacy or reduce the side effects of these therapies [4]. It also shows great therapeutic potential in the delivery of drugs for the treatment of non-tumor diseases [5]. However, their widespread use remains limited by disadvantages such as inherent toxicity, complex fabrication processes, and inadequate biocompatibility and safety [6]. As nanoparticles can often be recognized by the immune system in vivo, they can potentially cause strong adverse reactions. Despite the potential harm of generating anti-PEG antibodies, the US Food and Drug Administration (FDA) approved the use of polyethylene glycol (PEG)-conjugated liposomal doxorubicin for cancer treatment [7]. In addition, biological barriers such as the blood–brain barrier are also difficult barriers for nanoparticles to overcome, preventing them from effectively targeting certain tissues [8].

In recent years, exosomes, emerging nanoscale biological carriers, have attracted increased attention in the field of tumor therapy. Exosomes are extracellular vesicles (EVs) that can arise in normal or abnormal cells and mediate intercellular communication by translocating biologically active cargo such as nucleic acids, proteins, lipids, and metabolites to target cells, thereby regulating target cell and tissue functions [9]. Such lipid membrane-enclosed vesicles of approximately 30–150 nm in diameter, are commonly found in body fluids such as blood, urine, saliva, cerebrospinal fluid, and breast milk [10]. The detection of exosomes in body fluids for the early screening of diseases has become an emerging diagnostic field [11]. Almost all cell types can produce and release exosomes. Exosomes were initially recognized as a cellular waste disposal mechanism; however, in subsequent studies, they were found to play a mediating role in intercellular information exchange and material transport [12]. Due to their tiny size at the nanometer scale, exosomes are able to effectively evade macrophage phagocytosis while easily crossing the vessel wall and extracellular matrix. When exosomes are used as drug carriers, the drug is encapsulated within the exosome and subsequently transported through body fluids to the disease site, avoiding an immune response, and thus being rapidly cleared. Furthermore, it is considered a promising drug delivery vehicle because of its biocompatibility, natural targeting, and ease of modification [13]. In addition, exosomes can also serve as biomarkers for tumor diagnosis and prognosis evaluation. In this review, we summarize recent advances in the isolation, identification, drug loading, and modification of exosomes as drug carriers for tumor therapy alongside their application in tumor therapy. Basic knowledge of exosomes, such as their biogenesis, sources, and characterization methods, is also introduced herein. In addition, challenges related to the use of exosomes as drug delivery vehicles are discussed, along with future trends. This review provides a scientific basis for the application of exosome delivery systems in oncological therapy.

## Biogenesis and source

### Biogenesis

**Fig. 1:**
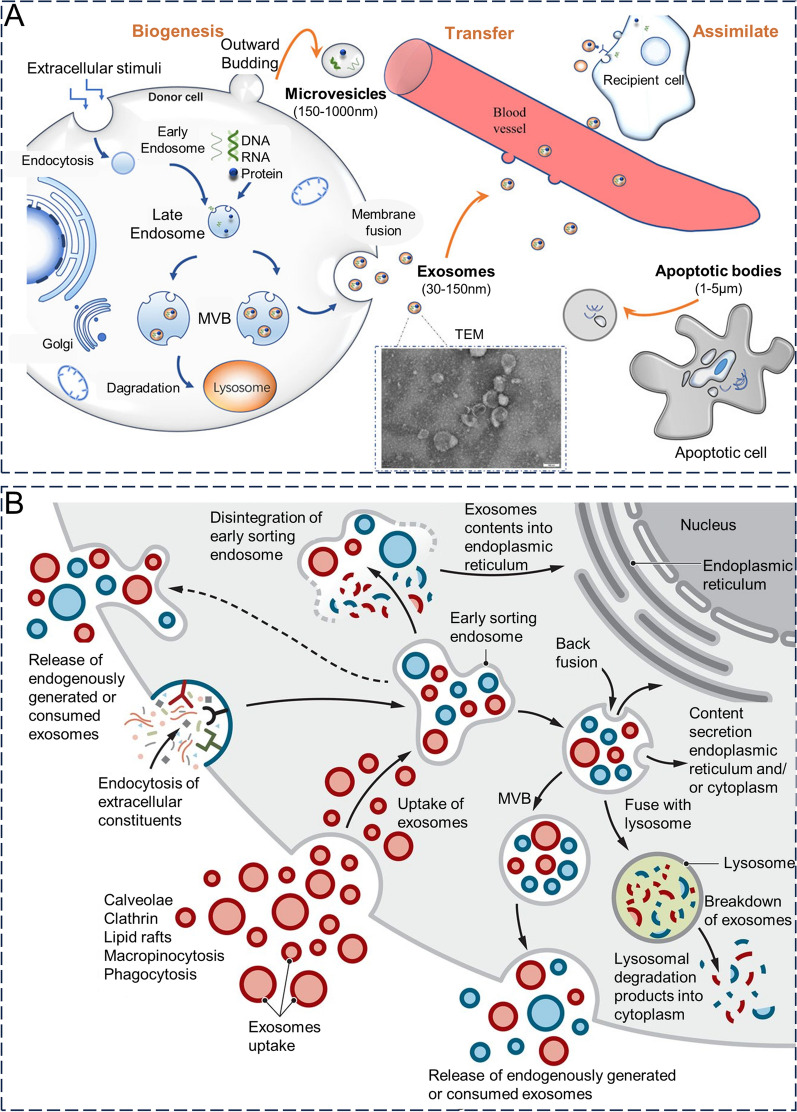
**A** Biogenesis of exosomes. Reproduced with permission. [17] Copyright 2022, Frontiers. **B** The intracellular fate of exosomes. Reproduced with permission. [12] Copyright 2020, American Association for the Advancement of Science

Exosome formation is believed to involve two plasma membrane invagination processes. Initially, the cytoplasmic membrane undergoes invagination to form an early endosome [14]. During this process, components of the cell membrane are captured and become part of the endosomal membrane. Subsequently, the early endosomes gradually mature into late endosomes. Subsequently, late endosomal membranes reverse outgrowth by forming multivesicular bodies (MVBs) with intraluminal vesicles (ILVs) [12]. After MVB formation, there are several possible destinations: (1) fusion with the plasma membrane where ILVs are released as exosomes outside of the cell.; (2) fusion with lysosomes, whose contents are degraded by hydrolytic enzymes capable of digesting complex macromolecules; (3) retention within the cell to become organelles of specific cell types (e.g., melanosomes in melanocytes); and (4) recirculation through the trans-Golgi network (TGN) (Fig. 1A) [15–17]. The factors determining the fate of MVBs remain unknown, whilst Rab GTPase is thought to be a determinant in the regulation of vesicular transport [14].

The tetraspanin family of proteins (e.g., CD9, CD63, CD81,and CD82), major histocompatibility complex (MHC), heat shock proteins (Hsp), transcription factors, cytoskeletal components, nucleic acids (genomic and mitochondrial DNA and RNA), and lipids are common loaders for exosomes (Fig. 1B) [7]. Among them, the family of four transmembrane proteins and heat shock proteins are often used as a marker of exosomes for extraction, isolation, and analytical identification. During exosome formation, the contents are selectively loaded through endocytic pathways of the cell membrane and cytoplasmic lysates. There are two primary pathways for the selective loading of proteins: the pathway involving the endosomal sorting complex required for transport (ESCRT) and the pathway that does not rely on ESCRT [18]. In the ESCRT-dependent pathway, four complex proteins (ESCRT-0, -I, -II, and -III) regulate exosome formation and transport, with ESCRT-0 mediating the recognition and sorting of ubiquitin-dependent loaders. Meanwhile, ESCRT-I and ESCRT-II mediate the inward budding of endosomal membranes, with ESCRT-III being responsible for endosomal vesicle excision, leading to its shedding into the endosomal lumen to form MVB [19–21]. Three main pathways have been proposed for non-dependent ESCRT in human embryonic kidney 293 cells: Ceramide-dependent, CD63-dependent, and non-dependent mechanisms [22]. The non-ESCRT-dependent pathway mediates vesicle outgrowth, movement, and fusion, primarily through the four transmembrane proteins, CD63, CD81, CD82, and CD9, alongside neutral sphingomyelinase 2 (nSMase2) [23]. Exosome biogenesis and cargo sorting can be regulated by oncoprotein—a transmembrane glycoprotein [24]. Depending on the type of exosome biogenesis process involved in ESCRT-dependent processes select ubiquitinated cytoplasmic proteins as cargo, whereas four-transmembrane protein-mediated ESCRT-independent selection does not require ubiquitination and instead selects a wide range of target proteins, including MHC receptors, metalloproteases, and β-linked proteins. Exosomal proteins primarily participate in antigen presentation, cell adhesion, maintenance of cell structure and motility, stress regulation,transcription,protein synthesis and transport, and membrane fusion. RNAs are also abundant in exosomes, including mRNA, miRNA, and non-coding RNAs such as piwi-interacting RNA (piRNA), small nucleolar RNAs (snoRNAs), long non-coding RNA (lncRNAs), tRNA, and Y RNA, among others [25]. The amount and proportion of RNAs in exosomes are different from those in the parental cells, suggesting that there may be a sorting mechanism used to select specific RNAs for exosomes [26]. In addition to RNA content,exosomes have been found containing genomic DNA and mitochondrial DNA that covers entire genome. Exosomes carry cell-free DNA from the fetus, whilst cell-free DNA may contribute to pregnancy complications by activating the inflammatory response; thus, it has been hypothesized that the blood of pregnant women could serve as a biomarker for pregnancy complications [27]. An in-depth understanding of the molecular mechanisms underlying the exosome formation process would facilitate the artificial regulation of its yield, protein composition, and envelope; however, the specific molecular mechanisms have not yet been fully elucidated.

### Source

**Fig. 2:**
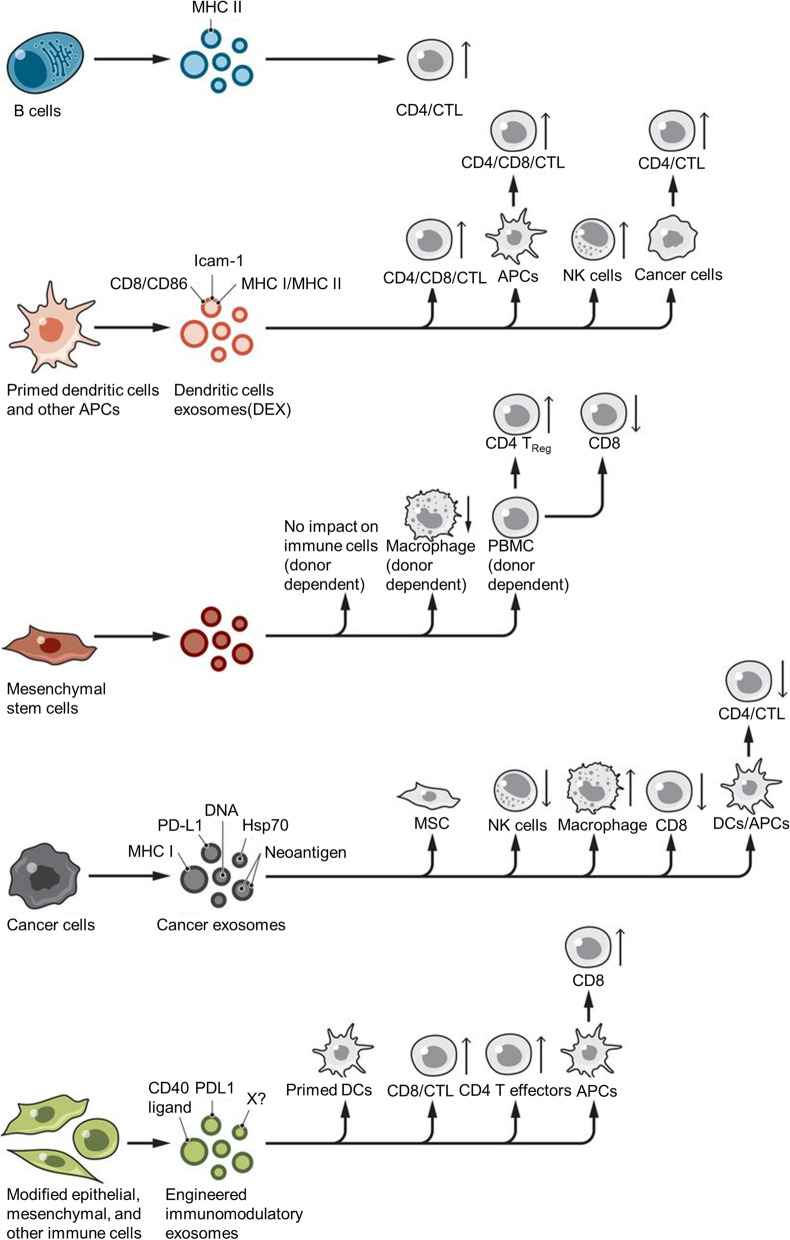
The donor cell of exosome mainly includes B cells, dendritic cells, mesenchymal stem cells, and tumor cells. Reproduced with permission. [12] Copyright 2020, American Association for the Advancement of Science

Most cell types, such as dendritic cells (DCs), macrophages, reticulocytes, mast cells, platelets, B cells, T cells, oligodendrocytes, and tumor cells, are capable of releasing exosomes (Fig. 2) [12]. Exosomes are composed of lipids (such as cholesterol, diglycerides, and sphingolipids), proteins (such as transmembrane transport-associated proteins, heat shock proteins, and TSPAN protein superfamily), and nucleic acids (such as DNA, miRNA, lncRNA, mRNA, and tRNA) from parental cells and also vary in the proportions of various components and surface markers, depending on the cellular origin [28]. This is because the composition of exosomes depends, to a large extent, on their cellular origin, and exosomes inherit specific biomolecules from parent cells which contributes to their heterogeneity. Moreover, different sources yield varying amounts, content, function, and drug loading capacity for exosomes leading to potential differences in therapeutic effects. Therefore, the selection of exosomes from the right source can help avoid side effects during drug delivery to a large extent [29].

#### Mesenchymal stem cells (MSCs)

Mesenchymal stem cells (MSCs) possess the typical attributes of stem cells, including self-renewal and the capacity for diverse differentiation in various directions. Additionally, they can undergo clonal expansion when exposed to specific in vitro stimuli, and the ability to differentiate into bone cells, adipocytes, and chondrocytes [30]. The most commonly used types of MSCs are bone marrow-derived MSCs (BMSC), adipose-derived MSCs (ADSC), and human umbilical cord-derived MSCs, which can be easily isolated from various tissues and then expanded in vitro. They possess the ability to adapt to the tumor microenvironment and secrete a large number of exosomes with strong paracrine activity. Compared with the other two sources of MSCs, adipose-derived MSCs have been studied the most in-depth. They can be obtained through subcutaneous fat aspiration, thus reducing issues surrounding collection such as pain and ethics [31]. Previous studies have shown that MSC-derived exosomes have immunomodulatory, bone regeneration-promoting, anti-inflammatory, anti-aging, and wound healing-promoting effects, alongside varying degrees of application in diseases such as myocardial infarction, acute kidney injury, and optic nerve injury [32]. Although both BMSC- and ADSC-derived exosomes exhibit potential therapeutic effects on wound healing and tissue regeneration, they play different roles as they primarily promote proliferation, whereas ADSC- EVs are highly associated with angiogenesis [33]. In addition, MSCs-derived exosomes have potential as delivery vehicles for antitumor drugs due to their tumor-targeting properties [34]. In a previous study by Li et al. [35], ADSC-EVs reduced tumor cell proliferation and migration by mediating enhanced tumor cell apoptosis, ultimately leading to tumor control in tumor-bearing mice. Additionally, the antitumor activity was further enhanced after ADSC-EVs were loaded with anti-oncogenic miRNA-16-5p. MSC-derived exosomes also protect encapsulated cargo from degradation when modified for cell type-specific targeting, these exosomes have the potential to become prospective tools for cell-free-based therapeutic approaches [31].

#### Macrophages

Macrophages, which are present in various parts of the body, perform crucial functions by regulating the immune system, inflammatory responses, antigen presentation, angiogenesis, and remodeling [36]. The tumor microenvironment (TME) is enriched with tumor-associated macrophages (TAM), which are key drivers of tumor progression, metastasis, and therapeutic resistance [37]. TAM can be categorized into two subtypes: Antitumor M1 and pro-tumor M2. M1-type macrophages, which have pro-inflammatory effects, are involved in the positive immune response and inhibit tumor growth. The primary mechanism underlying their ability to combat tumors lies in the presence of specific surface markers, namely major histocompatibility complex II (MHC-II), CD80, and CD86. Conversely, M2 type macrophages exhibit immunosuppressive properties by dampening immune responses and facilitating tumor growth while also exerting anti-inflammatory effects. Exosomes possess almost all properties of their origin cells; therefore, the functions of M1 and M2 macrophage exosomes differ [38]. M1 macrophage-derived exosomes are modified with aCD47 and aSIRPα on their membrane surface by click chemistry, which can specifically recognize CD47 on the membrane surface of tumor cells and SIRPα on the membrane surface of macrophages in vivo, thus blocking the "don't eat me" signaling between tumor cells and macrophages and allowing the active targeting of tumors. Simultaneously, antibody-coupled M1 exosomes were phenotypically shown to reprogram tumor-promoting M2 macrophages into antitumor M1 exosomes, which synergize with antibodies to exert antitumor effects [39]. Therefore, fully utilizing the characteristics of different subtypes of macrophage-derived exosomes is key for developing exosomal antitumor therapeutic strategies.

#### Dendritic cells (DCs)

Dendritic cells (DCs) are antigen-presenting cells that can activate T cells by recognizing tumor cell-associated antigens, resulting in an endogenous immune response from the host immune system against tumor cells [40]. Similarly to DCs, dendritic cell-derived exosomes (DEX) express major histocompatibility complex class I (MHC-I) and MHC-II, co-stimulatory molecules (CD86 and CD80), heat shock proteins, and adhesion molecules on the membrane surface that participate in antigen presentation and trigger CD4 + and CD8 + T cell activation [41]. DEX activation of CD4 + and CD8 + T cells after activation of DEX induces more effective antitumor immune responses in vivo through exosomal CD80 and endogenous IL-2 [42], which can then be used for the development of therapeutic vaccines for tumor immunotherapy. In addition, when used as a drug carrier, DEX has been shown to stimulate cytotoxic T cells in cancer therapy and inhibit tumor growth in animal models. Lu et al. [43] treated a mouse model of hepatocellular carcinoma (HCC) with exosomes derived from DCs expressing α-fetoprotein (AFP) (DEXAFP) and subsequently elicited a strong antigen-specific immune response and significant tumor growth inhibition, whilst the tumor microenvironment was also improved to some extent. In addition, DEX can overcome biological barriers such as the blood–brain barrier (BBB), making them more attractive for future drug delivery.

#### Tumor cells

Tumor cells can secrete tumor-derived exosomes (TEX) that contribute to various aspects of tumorigenesis including angiogenesis, proliferation inhibition, apoptosis promotion, growth and metastasis promotion, dormancy and chemoresistance induction, and immunosuppression [44], whilst playing an important role in tumorigenesis and development. Thus, TEX could be used as a target for tumor therapy. TEX carry tumor-related specific antigens alongside MHC-I-like molecules and can also deliver antigens to DCs to induce T cell-mediated immune responses against tumor cells [45]. Tetraspanin is specifically expressed on the surface of TEX, meaning that TEX is always preferentially homing and targeting its parental cells and being efficiently captured by parental cells [46], an advantage of using TEX as a drug carrier. Hepatocellular carcinoma cell-derived exosomes specifically express miR-103 and target the BBB connexin with enhanced BBB permeability, making it a potentially powerful tool for drug delivery in brain diseases [47]. In addition, many exosomes have been found in malignant effusions, and these ascite-derived exosomes could be used in cancer therapy. In phase I clinical trial for colorectal cancer immunotherapy using ascite-derived exosomes combined with granulocyte–macrophage colony-stimulating factor induced beneficial tumor-specific anti-tumor cytotoxic T lymphocyte responses [48]. Moreover, the distinctive surface antigens of TEX have the potential to mirror the characteristics of the cells from which they originate, allowing TEX to be used for monitoring disease progression and as a diagnostic marker. Notably, specific antigens on the surface of TEX derived from parental cancer cells have the potential to either accelerate tumor progression or cause immunosuppression, such as Tetraspanins, Urokinase plasminogen activator, Cathepsin D, and Vimentin, among others [49].

#### Plant-derived nanoparticles

In recent years, plant-derived nanoparticles (PDNPs) have been found to have a structure similar to that of mammalian exosomes, allowing interspecies communication between plants and animal cells and plant pathogens [50, 51], of which exosomes are one type. PDNPs contain various molecules, including bioactive metabolites, proteins, lipids, and nucleic acids [52]. PDNPs have also been shown to act as carriers to deliver bioactive and therapeutic molecules and can even cross mammalian biological barriers [53, 54]. Their high gastrointestinal stability and low production cost compared to mammalian cellular/humoral-derived exosomes give PDNPs a unique advantage in oral drug delivery [55, 56].

## Isolation, characterization, and storage

### Isolation and purification

**Fig. 3:**
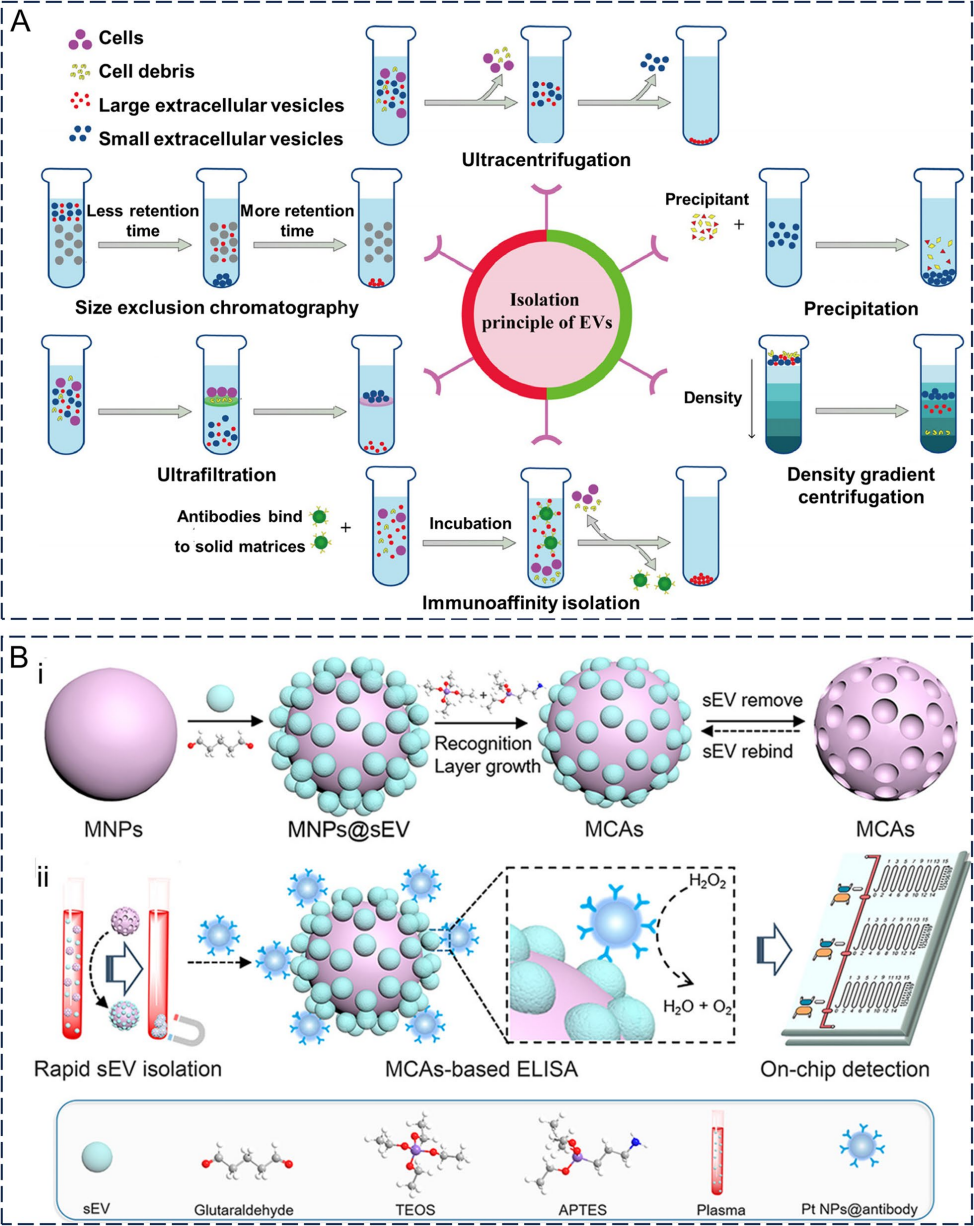
The main method of exosome isolation. **A** A common method for the isolation of exosomes from the source cells. Reproduced with permission. [58] Copyright 2022, Elsevier Ltd. **B** Flowchart of rapid isolation of exosomes based on magnetic colloid antibodies (MCA). (i) Preparation of MCA. (ii) Rapid isolation and analysis of MCA exosomes. Reproduced with permission. [90] Copyright 2021, American Chemical Society

The first step in applying exosomes as drug carriers, whether in body fluids or generated in cell culture, involves isolation and purification. Assessing the biological functions of exosomes is facilitated by achieving reproducible isolation and enrichment. Different isolation methods have been previously selected for different applications. Currently, the most commonly used methods include ultracentrifugation, size-based separation, polymer precipitation, and immunoaffinity chromatography (Fig. 3A) [57, 58]. However, the variability of exosomes in terms of their size, content, function, and origin complicates their separation. Most current techniques struggle to completely separate them from lipoproteins or extracellular vesicles with similar properties, resulting in low exosome purity. However, efficient enrichment of exosomes is essential for the downstream analysis of exosomes. Meanwhile, in recent years, combinations of two or more isolation and purification methods have provided reliable methods for efficient exosome isolation [59].

#### Ultracentrifugation

Ultracentrifugation (UC) is widely used and is the gold standard for exosome extraction and isolation [14]. UC is a method for separating exosomes from a sample using a high-speed centrifugal force. This method utilizes gradient centrifugation to isolate the desired fractions primarily by exploiting variances in size and density between the constituents of the initial solution, making it well-suited for separating substantial sample portions with notable disparities in sedimentation coefficients. UC is usually divided into three stages: (1) low-speed (~ 3 × 10^2^*g*) centrifugation to remove cells and apoptotic debris; (2) gradient increase in speed (centrifugal force of 2 × 10^2^*g*) to remove large extracellular vesicles; (2) ultracentrifugation (centrifugal force greater than 1 × 10^5^ g) to precipitate exosomes and wash them with phosphate-buffered salt solution to remove impurities. The yield and purity of the target exosomes can be influenced by various factors such as the duration of centrifugation, strength of centrifugal force, type of rotor used, and other parameters [17]. This technique is well-established, uncomplicated, cost-effective, does not necessitate labeled exosomes, and is suitable for handling substantial sample volumes. However, UC is also time-consuming (> 4 h) and has low extraction efficiency (which may cause losses of more than 40%), whilst some variability in purity may exist. In addition, high centrifugation forces may lead to exosome aggregation. Moreover, multiple centrifugations may damage the exosome structure, which is detrimental to downstream analyses [60].

#### Density gradient ultracentrifugation

The density gradient centrifugation method is derived from the ultracentrifugation method and further improves upon the efficiency of exosome separation. The principle of density gradient centrifugation is based on the fact that different extracellular components have different densities, whilst objects of a specific density are suspended in media of similar density [61]. This method usually involves the following steps: (1) Biocompatible media of different densities (e.g., sucrose or iodixanol) are placed in test tubes in a regular order of increasing densities from top to bottom. Notably, the choice of medium needs to cover the range of densities in the sample to be separated. (2) The sample to be separated is added to the test tube, and centrifuged for a prolonged period of time. (3) Finally, the exosomes, cell debris, and heteroproteins are suspended in a medium of equal density, and the separation of exosomes is completed [62]. Compared to ultracentrifugation, density gradient centrifugation provides exosomes with higher purity for downstream applications. Exosomes collected by UC are often contaminated with large amounts of lipoproteins and other biomolecules that co-precipitate with exosomes, whilst density gradient centrifugation can be used to further purify the resulting exosomes [63]. However, the application of density gradient centrifugation remains limited to some extent, owing to the very high equipment requirements of this method. Moreover, the density gradient centrifugation method, similar to the ultracentrifugation method, faces challenges in terms of varying degrees of damage to exosomes after ultra-high centrifugal forces [64].

#### Size exclusion chromatography

Size exclusion chromatography (SEC) is a size-based separation technique focused on differences in the hydrodynamic volumes among components in a sample. SEC has been previously used to separate exosomes from both prokaryotic and eukaryotic samples [57]. As the sample flows through the SEC column, smaller particles will enter the smaller gel pores within the column, increasing the passage distance and thus their outflow time. Conversely, larger particles cannot enter the gel pores and can only pass through the gaps between the porous gels, therefore allowing them to be eluted first. The main advantages of SEC are its improved separation efficiency and reproducibility, ability to maintain the structural integrity and uniform size of exosomes at lower pressures, lack of significant adverse effects on their biological properties, and ability to handle a wide range of sample types [65]. Currently, purification methods based on SEC principles, such as IZON ® qEV columns [66], Sepharose ® CL-2B columns [67], and Sephacryl ® S-400 columns [68], have been commercialized considering the rapid, simple, and low-cost application of SEC. However, limitations such as additional concentration steps, long separation times, need to equilibrate the column for each use, and small sample volumes that can be processed have limited the widespread use of SEC [69]. Furthermore, separated exosomes may also be contaminated with particles of similar size using SEC [70].

#### Ultrafiltration

Similar to SEC, ultrafiltration is a size-based separation technique. Ultrafiltration membranes with different pore sizes or molecular weight cut-off (MWCO) are used in the ultrafiltration method to selectively separate samples, often serving as an auxiliary separation method in exosome studies [71]. Generally, the size of the exosomes obtained by ultrafiltration depends on the pore sizes of the first and last membranes [72]. One previous study demonstrated that the highest separation efficiency was achieved when the pore size of the filtered membrane was 10 kDa [73]. Ultrafiltration is generally combined with different driving forces, such as charge, centrifugation [74], and pressure [75], to achieve the most efficient separation of exosomes. Ultrafiltration is simple to perform, does not require expensive specialist equipment, can be combined with other separation methods, and can be used for both small and large amounts of samples. However, problems such as nonspecific binding to the membrane, resulting in reduced recovery and potential destruction of exosomes by shear stress have limited the use of ultrafiltration [69].

#### Immunoaffinity capture

Immunoaffinity capture (IC) uses antibodies and ligands that specifically bind to form immune recognition sites and isolate exosomes. Considering the surface of exosomes is rich in specific membrane proteins such as CD9, CD63, CD81, CD82, and CD151, they can be targeted by specific antibodies as specific antigens [76]. By modifying the target proteins of these targets on the surface of magnetic beads, microfluidics, and chromatography matrices, the desired exosomes can be successfully obtained by capturing them using specific immunoconjugation between antibodies and antigens before washing them in the stationary phase. IC has high selectivity compared to other exosome separation methods, and can isolate exosomes containing specific membrane proteins whilst increasing the yield and enrichment rate by 10–15 times [77, 78]. IC can be used to isolate exosomes expressing a specific membrane protein, with the purity of the isolated exosomes being high, which can then be used for the detection and diagnosis of related diseases, such as CD326 + exosomes as a specific epithelial cancer-related biomarker [79]. On the contrary, affinity capture can make the isolated exosomes "biased" when there is no need to isolate specific exosomes, failing to cover both exosomes expressing/not expressing a certain membrane protein [80]. Although the specificity of this method is high, the separation efficiency is highly dependent on the specificity and accessibility of the antibodies, whilst most antibodies currently used for immunoaffinity capture are non-specific [81]. Additionally, the choice of eluent used to elute exosomes from the solid phase is also particularly important here. Non-neutral pH or non-physiological elution buffer conditions can have irreversible effects on exosomes, which can then interfere with downstream analyses [82]. In addition, IC is not suitable for the large-scale isolation of exosomes, as it requires higher-cost antibodies to process the samples [80].

#### Precipitation

Polymer precipitation is a size- and density-based separation method that uses polyethylene glycol (PEG) as a medium to harvest exosomes under centrifugal conditions by reducing their solubility. Precipitation was initially employed to isolate viruses but later showed good separation efficiency when purifying exosomes [83]. This method is relatively simple to perform, requires a short analysis time, can be integrated with existing clinical techniques, and is suitable for large sample volumes. However, exosomes isolated using this method are susceptible to polymer contamination, resulting in low purity and recovery, whilst this technique has limitations as it is difficult to remove polymers that may interfere with subsequent functional experimental analysis [84]. The Invitrogen kit was developed based on precipitation-isolated exosomes contaminated with PEG, although a negative effect on cell proliferation was observed when tumor cells were treated with the exosomes. The presence of PEG may be a possible reason for the toxicity of exosomes isolated using the Invitrogen kit. Precipitation may require an additional washing step during application, but this may increase its time cost, as well as reduce the yield [70]. Alternatively, Protein–Organic Solvent Precipitation (PROSPR) [85] and charge-based precipitation [86] are available options. PROSPR mainly uses organic solvents to precipitate soluble proteins such that exosomes are retained in the supernatant and further concentrated to obtain exosomes [85]. Meanwhile, charge-based precipitation relies on the combination of negatively charged exosomes with positively charged particles (e.g., fish sperm proteins) as a method of isolating exosomes [86].

#### Microfluidics-based methods for exosome purification

Microfluidics is a high-throughput, sensitive, and controllable exosome separation method that can be integrated alongside other methods to improve the efficiency and purity of exosome separation [87]. It divides the sample into tiny streams through channels on a chip with dimensions of tens to hundreds of microns, before combining size (hydrodynamic focusing, viscoelastic separation, deterministic lateral displacement, etc.) [88], immunoaffinity [89], and kinetics (e.g., magnetic (Fig. 3B) [90], electric [91], and acoustic [92] field) to separate exosomes from the microfluidic streams. In addition, sample pre-treatment, exosome separation, and in situ detection and analysis can all be integrated into a single microfluidic chip to simplify and automate the steps from exosome separation to analysis [93]. Furthermore, two or more combinations of different principles will give more functionality to the microfluidic device. Wang et al. [94] modified CD63 aptamer onto the surface of magnetic beads via a light-responsive group-nitrobenzyl, and the modified beads selectively bound CD63 + exosomes, which were then isolated under the action of an external magnetic field. The isolated exosomes must be further separated from the magnetic beads, whilst the captured exosomes are exposed to UV light at approximately 365 nm, where the photoresponsive group -nitrobenzyl is effectively cleaved; the purification of exosomes is achieved by removing these magnetic beads. The use of this photoresponsive group allows spatial and temporal control during exosome isolation whilst avoiding irreversible damage to exosomes from non-physiological eluates. Compared with conventional isolation methods, microfluidics allows for the more efficient acquisition of exosomes using smaller sample volumes and less time, while also enabling the isolation of specific subtypes of exosomes.

### Characterization

**Fig. 4:**
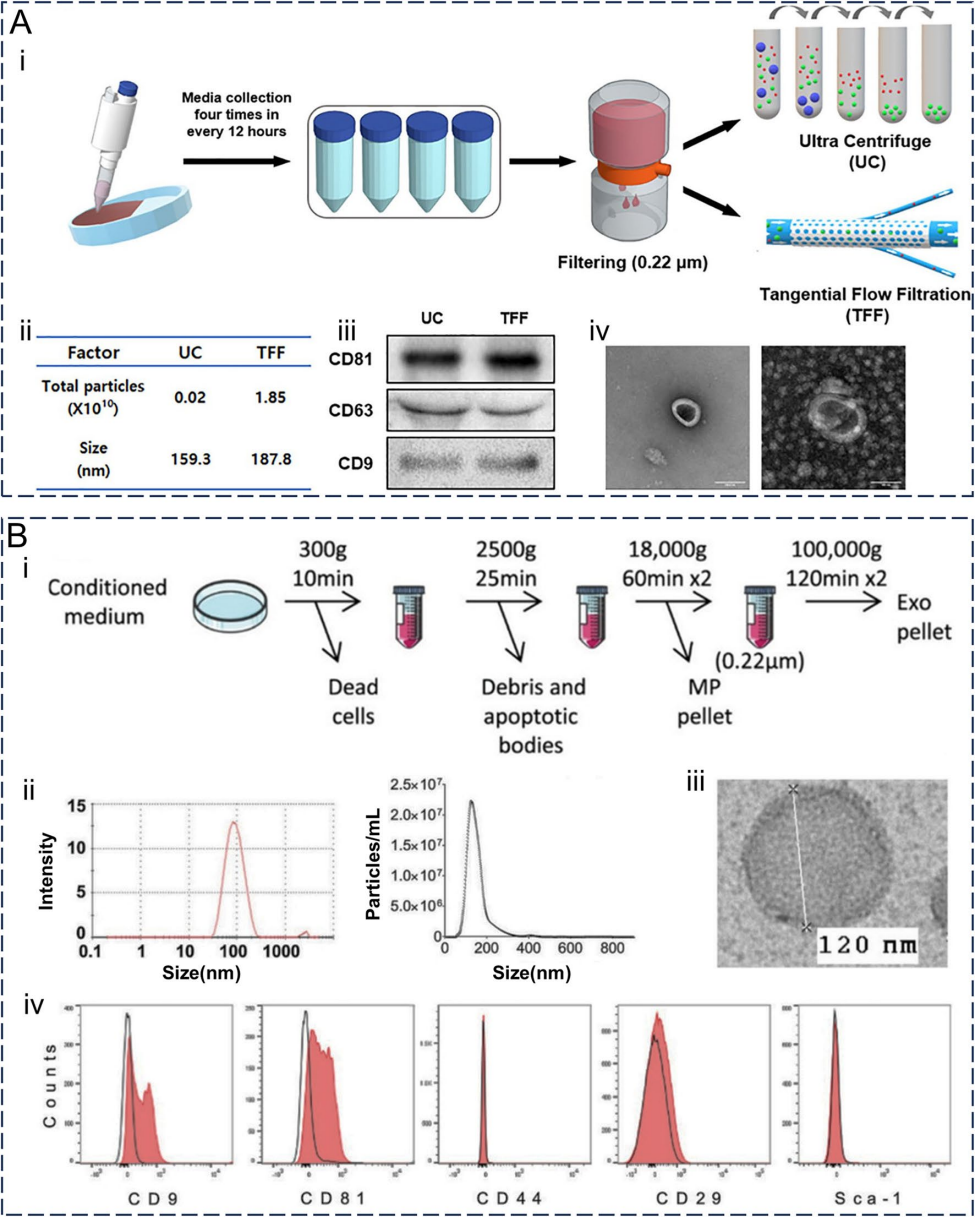
The main concerned properties and common methods in the characterization of exosomes. **A** Different performance of different separation methods under the same representation method. (i) MSC-derived exosomes were isolated by UC and TFF; (ii) Nanoparticle Tracking Analysis was used to measure particle size and number. (iii) Western bolt testing surface markers; (iv) Exosomes under transmission electron microscopy (left: UC, right: TFF, scale bar: 100 nm). Reproduced with permission. [75] Copyright 2021, SAGE Publications Ltd. **B** Characterization of mouse bone marrow-derived exosomes. (i) Flow chart of exosome isolation; (ii) Particle size and particle number were determined by dynamic light scattering (DLS) and nano-tracking analysis (NTA), respectively; (iii) Exosomes under transmission electron microscopy; (iv) Flow cytometry was used to detect surface markers. Reproduced with permission. [97] Copyright 2017, Springer Nature Ltd.

Extracted and purified exosomes require further validation, with characterization presenting an important tool for verifying the effectiveness of their extraction and providing a material basis for their application. Exosome characterization should include protein blot validation of specific markers alongside at least two methods to characterize individual exosomes [95]. Currently, quantitative methods for exosomes need to be used in combination and only indirectly reflect the number of exosomes. The commonly used methods for this are the total protein amount and total particle number. Additionally, qualitative characterization requires imaging techniques and biophysical characterization. To demonstrate that exosomes are closed vesicles with a lipid bilayer structure, at least one transmembrane/lipid-binding protein and one cytoplasmic protein need to be characterized. Furthermore, proteins that are most likely to contaminate exosomes during isolation must be characterized and used to control their purity. The physical and biological properties of exosomes and their practical applications can be characterized using appropriate methods (Fig. 4A) [75]. For example, electron microscopy (scanning electron microscopy, projection electron microscopy, and atomic force microscopy) can be used to demonstrate surface morphology information, whilst also using dynamic light scattering to measure exosome size and zeta potential distribution, and nanoparticle tracking analysis to determine particle concentration and size distribution. For exosomal proteins, enzyme-linked immunosorbent assays, protein blotting, flow cytometry, chromatography, and mass spectrometry can be used. Meanwhile, for exosomal RNA and DNA, real-time fluorescence quantitative PCR, digital PCR, and NGS sequencing can be used, whilst mass spectrometry techniques are often employed for exosomal lipids (Fig. 4B) [14, 96, 97]. In recent years, new protein detection methods (such as olorimetric detection, fluorescence detection, electrochemical detection, surface plasmon resonance detection, surface-enhanced Raman scattering, and CRISPR/Cas system-assisted detection) and nucleic acid detection methods (such as single vesicle analysis, thermophoretic detection, and CRISPR/Cas-assisted detection) have emerged, whilst the rapid development of exosome detection technologies has facilitated their use in diagnosis [11]. Notably, exosomes may also change their physical or biological properties during characterization. In conclusion, comprehensive characterization of exosomes helps determine their properties from multiple perspectives whilst providing strong support for their subsequent application.

### Storage

Exosomes that are not used immediately after isolation must be properly stored to protect their biological activity and facilitate transport and clinical applications. Cryopreservation, freeze-drying, and spray-drying serve as the primary storage techniques employed for exosomes. Cryopreservation is a storage method that involves lowering the temperature below the threshold required for biochemical reactions to ensure the functional stability of biological particles. Typically, this technique is implemented at temperatures of 4 °C, − 20 °C, and − 80 °C. Compared to freshly isolated exosomes, different storage temperatures and times have different degrees of impact on the number, size, stability, in vivo distribution, and other properties of exosomes. Considering subsequent functional studies, storage at 4 °C or − 20 °C is generally recommended for short-term storage with − 80 °C used for long-term [98]. Notably, direct cryopreservation is prone to irreversible damaging exosomes, which is mainly related to an imbalance in osmotic interactions during freezing and the occurrence of frozen crystals within organic particles. Therefore, exosomes are selectively cryopreserved by the addition of one or more appropriate concentrations of cryopreservative agents. Cryopreservatives are classified as osmotic (e.g., dimethyl sulfoxide and ethylene glycol) or non-osmotic (e.g., alginate and sucrose). In addition, exosomes should avoid undergoing repeated freeze–thaw cycles [99]. Freeze-drying and spray-drying, on the other hand, are based on the principle that water in exosomal solutions is evaporated by freezing in a vacuum through direct sublimation of ice or by atomization followed by evaporation in high temperatures. Similar to cryopreservation, freeze-drying requires the addition of cryoprotectants to maximize the preservation of the morphology and pharmacokinetics of exosomes from being altered. Furthermore, the protein content of freeze-dried exosomes stored at room temperature is similar to that of exosomes kept at − 80 °C [100]. Unlike cryopreservation and freeze-drying, the key factors impacting exosome stability during spray-drying are the pressure used for atomization and the temperature at which they exit. Although both freeze- and spray-drying yield exosome powders, spray-drying is a continuous process that allows for adjustment of powder particle size [101].

## Loading strategy

**Fig. 5:**
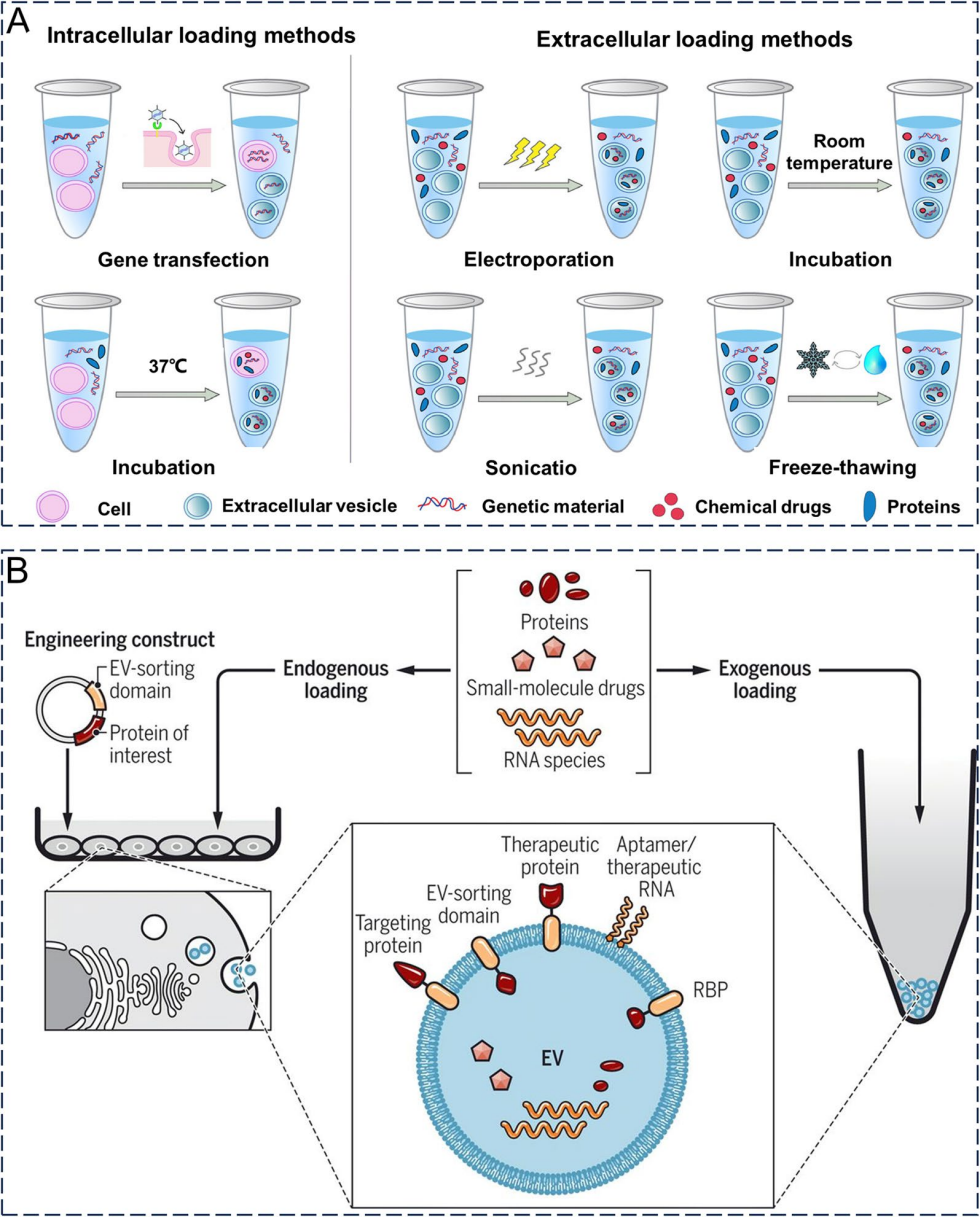
The main strategy for cargo loading into exosomes. **A** Loading methods are mainly divided into presecretory loading (left, intracellular loading) and postsecretory loading (right, extracellular loading). Reproduced with permission. [58] Copyright 2022, Elsevier Ltd. **B** Therapeutic agents such as proteins, small-molecule drugs and RNA species can be loaded onto the surface or inside of exosomes before or after secretion, or donor cells can be engineered to express molecules of interest and then secrete exosomes loaded with such molecules. Reproduced with permission. [10] Copyright 2019, Association for the Advancement of Science

Nucleic acids (e.g., short interfering RNAs and antisense oligonucleotides), proteins, and drugs (e.g., chemotherapeutic agents and immunomodulators) can be loaded into exosomes as cores. Loading these substances into exosomes is a key aspect in the development of exosomal drug delivery systems. Additionally, the following factors must be considered here: (1) achieving better encapsulation or loading efficiency, (2) maintaining the structural integrity of the exosome, and (3) maintaining drug activity. The main therapeutic agent loading strategies for exosomes include pre and post-secretory loading (Fig. 5A, B) [10, 58, 102]. Pre-secretory drug loading involves loading therapeutic agents directly into parental cells or modifying parental cell genes to secrete engineered exosomes. Furthermore, presecretory drug delivery consists of two main methods: coincubation and genetic modification. Coincubation usually involves the co-culture of parental cells with a therapeutic agent (e.g., paclitaxel [103], gemcitabine [104], or adriamycin [105]), allowing the therapeutic agent to cross the cell membrane and enter the cytoplasm. In contrast, genetic modification is performed by transfection and genetic modification of parental cells to overexpress the desired therapeutic agent (e.g., RNA or protein) [106]. The therapeutic substance within the cytoplasm is segregated into exosomes through either active or passive mechanisms, subsequently released from the cell alongside these exosomes. The extraction technique employed ensures the acquisition of suitable exosomes [107]. This method involves only the treatment and modification of parental cells, whereas the extracted exosomes are relatively untreated. The primary advantage is that the integrity and functionality of exosomes are better preserved, although drug-loading efficiency is difficult to control and is often low.

Post-secretory loading is the most common method of exosome loading and can be further divided into active and passive loading. The general procedure for this first isolating and purifying the exosomes, before properly loading the therapeutic agent. Electroporation and sonication are the most commonly used active loading methods, whereas the other methods involve repeated freeze-thawing and extrusion [2]. The advantages of this method are the relative simplicity and relatively high loading efficiency. Nonetheless, the loading procedure could potentially undermine the exosomes' soundness, necessitating further refinement measures to eliminate any unloaded freight [108]. Incubation, which is a passive loading method, has also been widely used in cancer research. This method relies mainly on the principle of passive diffusion, in which hydrophobic drugs pass through the exosomal membrane down a concentration gradient without harming the integrity of the exosomal membrane. However, the limitations of incubation are also obvious, namely, the low drug-loading rate. The drug loading efficiency of exosomes may be influenced by the drug's hydrophobicity, method of drug loading, and lipid composition of exosomes [2]. Therefore, in practical applications, a suitable drug-loading method must be selected based on the physicochemical properties of the drug. The principles, advantages, and limitations of the exosome loading methods are summarized in Table 1.

**Table 1:** The principles, advantages, and limitations of the exosome loading methods

Loading strategy	Principle	Advantage	Disadvantage	References
Coincubation	By incubating the exosome or donor cell directly with the drug (typically a lipid-soluble small molecule), the drug can enter the exosome or donor cell by diffusion along a concentration gradient	Simple operation No additional active substance is required	Only fat-soluble drugs can be loaded Low loading efficiency	[109]
Extrusion	The exosomes were mixed with the drug, and the mixture was then loaded into a syringe-based lipid extruder with a 100-to 400-nm porous membrane at a controlled temperature. During extrusion, the exosome membrane is ruptured and violently mixed with the drug	High yield Suitable for mass production	The properties of the membrane (e.g., zeta potential) and membrane protein structure may be altered Potentially cytotoxic	[110]
Sonication	The exosomes were incubated with drug molecules, etc., and then the membrane of the exosomes was deformed by sonication and the mechanical shear force of the sonication probe to make the drug enter the exosomes	High drug loading efficiency	The drug may attach to the membrane surface Not effective against hydrophobic drugs	[111]
Electroporation	An electric field is applied to the exosome suspended in a conductive solution, and the current interferes with the phospholipid bilayer of the exosome, creating temporary holes in the membrane through which drugs, etc., can diffuse into the interior of the exosome	No chemical reagents are introduced High efficiency in loading hydrophilic drugs (e.g., DNA and RNA)	May result in the introduction of drugs that fuse with the cell's own components	[112]

## Surface functionalization strategy

Natural exosomes, when used in tumor therapy, act as drug carriers to increase the effective concentration of drugs, although sometimes suffer from short half-lives and poor targeting, which limits their usefulness and application [113]. Modification of the exosome surface can reduce these limitations to some extent. Generally, exosome surface proteins serve as anchoring mechanisms or affinity markers, enabling the attachment of desired protein or peptide components through various techniques such as chemical modification, physical manipulation, or gene editing [99]. Currently, modification strategies for exosomes can be divided into two main types: pre-secretory cellular-level modifications (e.g., genetic engineering, metabolic engineering, and direct parent cell membrane labeling) and post-secretory exosome-level modifications. Post-secretory exosome-level modifications include click chemistry, multivalent electrostatic interactions, ligand-receptor interactions, hydrophobic interaction/membrane engineering, aptamer-based surface modification, and modification by anchoring the CP05 peptide [114]. Exosome-level modifications are usually performed after the isolation and extraction of exosomes. Modified exosomes exhibit improved passive/active targeting, cellular uptake, and immune evasion capabilities in terms of function compared to natural exosomes. In addition, specifically modified exosomes can respond to exogenous stimuli to allow the slow release of a loaded drug.

### Gene engineering

**Fig. 6:**
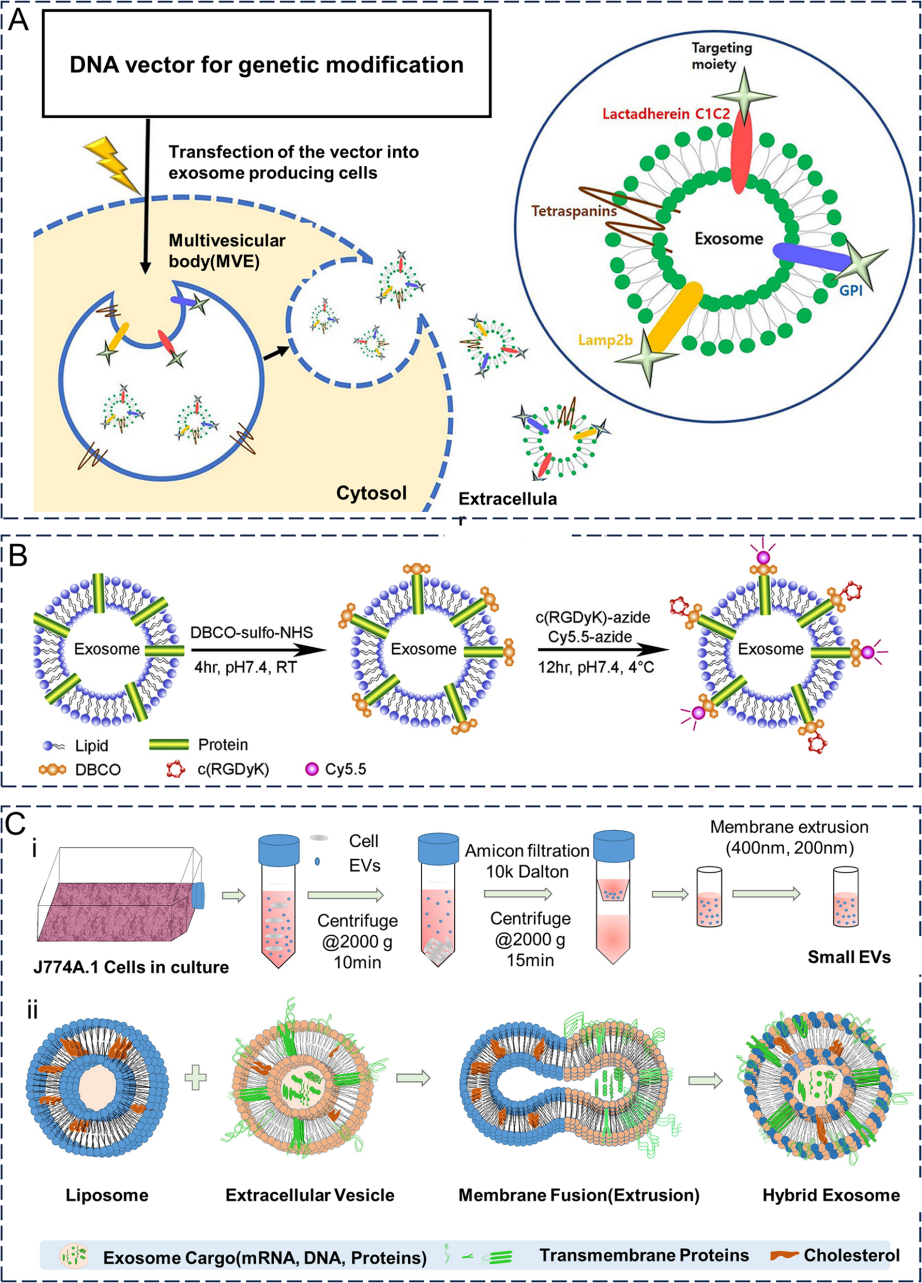
Strategies for surface functionalization of exosomes. **A** Exosome-producing cells are genetically modified to secrete exosomes expressing targeted proteins for therapeutic or targeted effects. Reproduced with permission. [115] Copyright 2021, Springer Nature. **B** Flow chart of c(RGDyK) and Cy5.5 coupled to the surface of exosomes. Reproduced with permission. [120] Copyright 2017, Elsevier Ltd. **C** Hybrid exosomes were obtained by co-extrusion of exosomes isolated from J774A.1 and liposomes. Reproduced with permission. [129] Copyright 2019, Elsevier Ltd.

The principle of genetic engineering is to genetically modify parental cells with an exosomal secretory capacity to express the target protein on the surface of their cell membranes, which in turn is stably displayed on the surface of exosomes secreted by the cell. This method takes advantage of the inherent protein expression mechanism of the cell and the natural biogenesis of exosomes, thereby facilitating the preservation of the natural conformation and function of the expressed target protein or peptide (Fig. 6A) [115]. This strategy generally utilizes the highly expressed membrane proteins of exosomes as anchor sites for target proteins. Currently, the most commonly used anchor sites of exosomes include tetraspanins, lysosome-associated membrane glycoprotein 2b (Lamp2b), lactamycin (LA), and glyco-sylphosphatidylinositol (GPI), among others [116]. The cellular level-based modification strategy can effectively modify the targeted ligands on exosomes; however, it is relatively time-consuming as it involves transfection of the parent cell and is less suitable for the modification of exosomes from patient body fluids. Longatti et al. [117] genetically engineered HEK293 cells to express a single-stranded variable fragment (scFv) structural domain on their surface, which was then fused to the C1C2 structural domain of lactamycin. The scFv structural domain has an affinity for the cell surface receptor ERBB2 (Her2). The experimental results demonstrated that HEK293 cells secreted exosomes that not only expressed the scFv structural domain on their surface but also targeted Her2. Compared to natural exosomes, exosomes expressing a high affinity for Her2 showed selective uptake in tumor cells at high Her2 expression levels, with uptake rates approximately 2–3 times higher than those of natural exosomes. In addition, exosomes can be constructed as multifunctional delivery vectors using genetic engineering. Wang et al. [118] transfected Lamp1b, tyrosine, and iRGD peptide genes into HEK293 cells using a lentivirus and obtained exosomes expressing Lamp1b, tyrosine, and iRGD. These exosomes were then loaded with DOX, with I^131^ being modified at the tyrosine site on the exosome surface. The drug-loaded exosomes obtained through this series of steps exhibited excellent tumor-targeting ability and tumor growth inhibition, both in vitro and in vivo. The combination of chemotherapy and radiotherapy, as well as their combined antitumor effects, also showed good biosafety.

### Chemical modification

Chemical modification involves the attachment of target proteins to the exosome surface via chemical reactions using lipid-binding proteins, membrane-bound proteins, or lipid–lipid interactions. The chemical modification strategy is mild, efficient, and time-consuming, although the modification conditions need to be strictly controlled to prevent exosomes from being exposed to too many reagents, too high a temperature or pressure, etc., which may then damage the exosomal membrane and the structural and functional integrity of the protein [116]. Currently, this modification strategy can be divided into categories of covalent and noncovalent bonds.

The most commonly used covalent binding method is click chemistry, a class of chemical coupling reactions that occur under aqueous, buffered, and physiological conditions that produce irreversible bonds. To modify exosomal membranes using click chemistry, the conversion of exosomal amine groups to alkynes must be ensured; that is, the targeting ligands must be coupled to the exosomal membranes using covalent bonds formed between azides and alkynes [119]. Based on this strategy, orthogonal chemistry (click chemistry)-targeted exosomes have been developed. Tian et al. [120] first combined a reactive dibenzylcyclootyne (DBCO) group with amine-containing molecules on exosomes from MSCs (Fig. 6B). The cyclo(Arg-Gly-Asp-D-Tyr-Lys) peptide [c(RGDyK)] was modified with the azide group to lysine and the azide group on c(RGDyK) was then bonded to the amine group by click chemistry, thus modifying c(RGDyK) to the exosome surface. The c(RGDyK)-conjugated exosomes (cRGD-Exo) then targeted ischemic brain lesion regions in a mouse model of ischemia–reperfusion. Subsequently, curcumin-loaded cRGD-Exos inhibited inflammatory responses and apoptosis in the lesioned region more effectively than curcumin or exosomes alone in a similar mouse model. In contrast, click chemistry could be used to enhance the immune response of tumor cells to exosomes. In most tumors, signal regulatory protein α (SIRPα) on the surface of macrophages often interacts with CD47 (a "don't eat me" signal) on the surface of tumor cells, limiting the ability of macrophages to phagocytose tumor cells. Koh et al. [121] modified the SIRP-α variant on the exosome surface using click chemistry to form SIRP-α-exosomes. By interfering with CD47-SIRP-α interaction, SIRP-α-exosomes enhance macrophage phagocytosis whilst increasing the number of tumor cells phagocytosis. In a homologous mouse cancer model, SIRPα-exosomes inhibited tumor growth whilst enhancing T-cell infiltration in mice. In addition, metabolic glycan engineering allows the direct modification of living cells with substrates under laboratory conditions, which can be used for in vitro or in vivo click chemistry [122]. Compared with traditional covalent binding methods, click chemistry is simple, efficient, and does not require toxic catalysts. Therefore, advances in "click chemistry" technology are important for the rapid mass production of engineered exosomes [123]. However, the modification of click chemistry may affect the functioning of other membrane proteins in the exosome membrane, such as cell-binding proteins, proteins that mediate immune escape, and intracellular transport pathways. Therefore, this strategy also requires precise control of reaction conditions, such as the ratio of targeting ligands to exosomes. Additionally, this approach is not usually applicable to specific exosomal amine groups [2].

Noncovalent binding involves the modification of targeting ligands to exosome membranes through noncovalent bonds, including charge and hydrophobic interactions. Compared to the covalent coupling strategy, the modification conditions of this strategy are relatively mild, although the binding strength is weaker. Qi et al. [124] first bound transferrin to the corresponding receptor on the surface of serum exosomes before binding the superparamagnetic nanoparticle to transferrin, thus obtaining superparamagnetic behavior-exhibiting exosomes that were more responsive to external magnetic fields than free superparamagnetic nanoparticles. In vivo, modified exosomes target tumor cells and inhibit their growth in response to an external magnetic field. Aminoethyl ethanolamine (AA) binds to sigma receptors that are highly expressed on the surface of tumor cells. In a previous study, aminoethyl anilamide-polyethylene glycol (AA-PEG) was integrated into the exosome membrane using sonication before being loaded with paclitaxel. The resulting exosomes could target sigma receptor-expressing tumor cells and inhibit their growth. Compared to exosomes loaded with paclitaxel alone, this new exosome had a stronger therapeutic effect on lung metastases [125]. In addition, polyethylene-glycolized exosomes showed enhanced cell specificity and circulation time [126]. Thus, it is evident that membrane integration of targeting ligands on exosomes could be achieved through lipidation or hydrophobic modification; however, the efficiency of targeting ligand introduction and binding stability still require further optimization.

Chemically modified exosomes can acquire new functions or enhance their existing ones, such as targeting cell adhesion molecules (iRGD, CRGDKGPDC; LFA1, lymphocyte function-associated antigen-1), cell receptors (AA-PEG, aminoethylamide polyethylene glycol; GE11, amino acid sequence YHWYGYTPQNVI; RVG, rabies virus glycoprotein), antigen presentation (GALA, a pH-sensitive fusion peptide), and T cells (major histocompatibility complex). For example, surface modifications based on the CP05 peptide provide a tool for enhancing exosome targeting and therapeutic functions, which can be used for in vivo detection and drug delivery in cancer therapy. The combination of CP05 with the exosome surface protein CD63 enhances exosome targeting in enriched target organs/target tissues, such as muscle, the brain, and subcutaneous tumors. Moreover, modification of CP05 did not cause significant changes in the physiological properties, structure, or surface molecules of exosomes [127].

### Hybrid membrane engineering

Instead of directly modifying the exosome membranes, hybrid membrane engineering fuses liposomes containing membrane-functionalized molecules (ligands, antibodies, and PEGs) with exosomes or parental cells to form hybrid exosomes, thereby functionalizing the exosome membranes (Fig. 6C) [128, 129]. Macrophage-derived exosomes have been fused with cationic, anionic, fluorescently labeled, and polyethylene glycolized liposomes by repeated freeze-thawing to form hybrid exosomes, whose interaction with cells in vivo was then altered, likely due to the altered lipid composition of the hybrid exosomes [130]. Li et al. [131] co-incubated liposomes containing fluorescence and azide with parental cells and subsequently isolated exosomes, including fluorophores, lipids, azides, and targeted peptides, were coupled by copper-free click chemistry. In addition, hybrid membrane engineering can add smart response functions to exosomes in combination with other modification methods. Lv et al. [132] fused genetically engineered exosomes expressing CD47 with heat-sensitive liposomes to treat metastatic peritoneal carcinomas (120 mPC). Under hypothermic conditions of hyperthermic intraperitoneal chemotherapy (HIPEC), transvenously injected hybrid exosomes accumulated in tumors of mice in vivo, showing significant antitumor effects when loaded with GM-CSF or doxorubicin (DTX). Compared to drug-loaded hybrid exosomes alone, drug-loaded hybrid exosomes in combination with HIPEC have stronger antitumor effects and can achieve temporal and spatial targeting of therapeutic agents to tumors in vivo. In addition, hybrid exosomes can be used to deliver the CRISPR/Cas9 system to MSCs, which cannot be transfected with liposomes alone [133].

## In vivo characteristics

### Mechanisms of uptake

Exosomes are released into the extracellular environment by the cytosol and rely on surface proteins that bind to membrane receptors for attachment to specific target cells. Exosome contents are released into the cytoplasm of target cells via integrins, tetraspanins, and intercellular adhesion molecules [134]. The pathways of exosome internalization by target cells include Clathrin/caveolin-mediated endocytosis, lipid raft-mediated uptake via lipid rafts, macropinocytosis, direct fusion with the cell membrane, and phagocytosis [135]. Exosome surface proteins help detect the process of exosome internalization by target cells [136]. Exosomal contents can trigger genomic, proteomic, and epigenetic alterations in cells whilst playing regulatory roles in intercellular signaling, organ development, and physiological functions. Notably, the uptake of exosomes does not equate to their function in cells, and there remains a possibility of degradation by lysosomes [13].

### Biodistribution and pharmacokinetics

Similar to other nanovesicles, factors like particle size, surface charge, protein profile, lipid bilayer composition, and dosage can affect the tissue distribution of exosomes. Previous studies using radiotracers have demonstrated that unmodified tumor-derived exosomes administered intravenously have a short half-life of approximately 2 min in circulation [137]. Within an hour after injection, accumulation occurs primarily in the liver and spleen due to rapid uptake by these organs. After 24 h, significant accumulation is observed in the liver and spleen along with some presence in the lungs and kidneys [113]. The mononuclear phagocytic system (MPS) plays a major role in clearing exosomes from circulation. Although exosomes possess a slightly negative surface charge which may reduce MPS-mediated clearance compared to positively charged nanoparticles (NPs) [138], their circulation time could be adversely affected by phosphatidylserine (PS), a negatively charged lipid present on their surface [139]. Appropriate surface modifications could improve the rapid clearance of exosomes from somatic circulation.

**Fig. 7:**
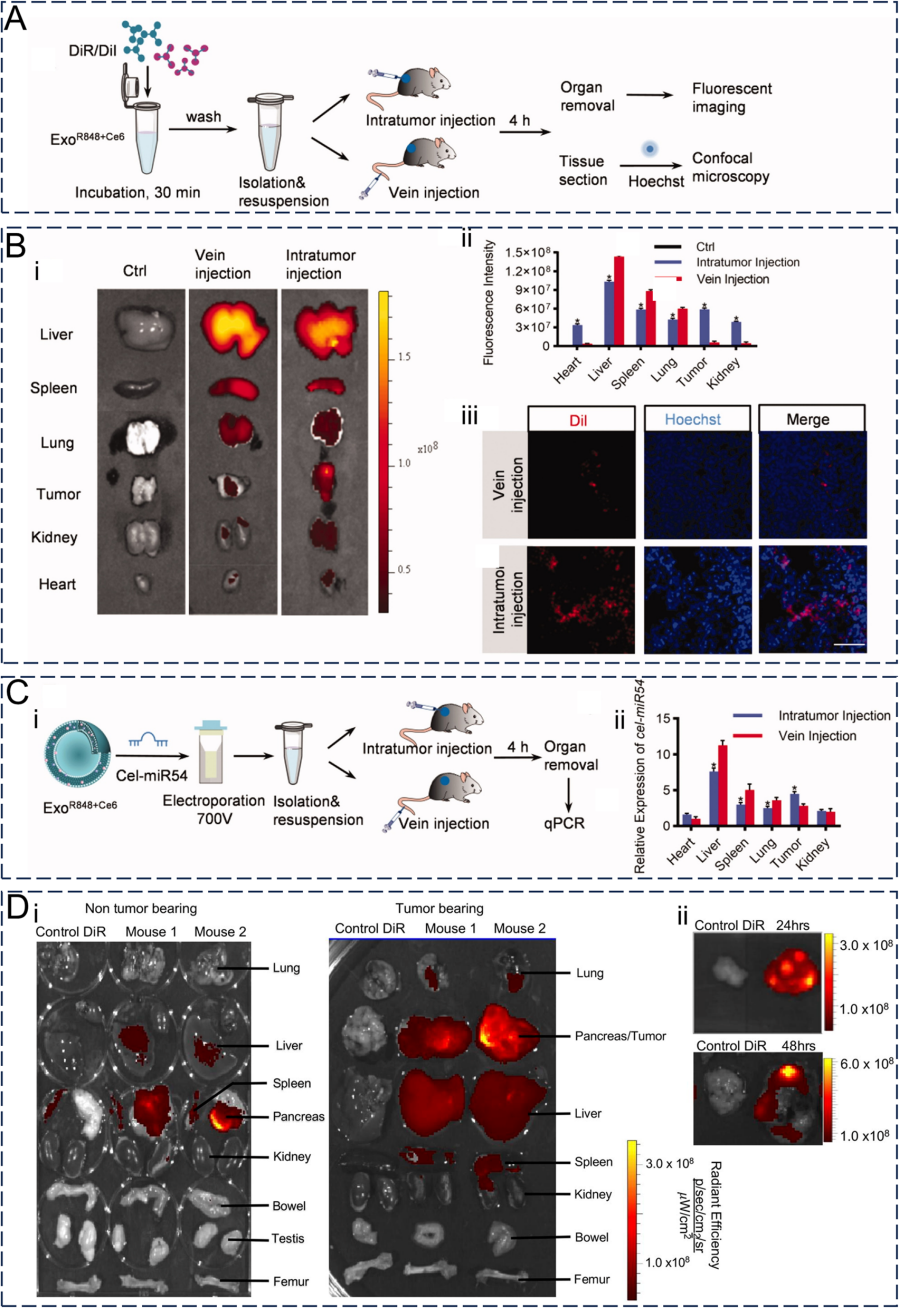
Distribution characteristics and pharmacokinetics of exosomes in vivo. **A** DiR/DiI-labeled exosomes were injected intraperitoneally or intravenously into mice to monitor the distribution of exosomes in vivo. **B** Ex vivo fluorescence images (i) and quantification plots (ii) of vital organs and tumor sites in mice after intraperitoneal or intravenous injection of DiR/DiI-labeled exosomes. (iii) Distribution of DiR/DiI-labeled exosomes at tumor sites. Scale bar = 100 μm. **C** After the exosomes loaded with cel-miR54 were injected intraperitoneally or intravenously into mice, (i) the distribution of exosomes in vivo and (ii) the expression of cel-miR54 in important organs and tumor sites in mice. Reproduced with permission. [140] Copyright 2022, Informa UK Limited. **D** Distribution of DiR-labeled exosomes in vital organs of tumor-bearing and non-tumor-bearing mice (i) and (ii) accumulation of exosomes at tumor sites 24 or 48 h after intraperitoneal injection. Reproduced with permission. [145] Copyright 2020, Wiley

The route of administration also affects the tissue distribution of exosomes to some extent (Fig. 7A–C) [140]. The routes of exosome administration can be divided into systemic and local. Systemic routes of administration include intravenous, oral, intraperitoneal, and nasal routes, with intravenous injection being the most common. Meanwhile, local routes of administration are relatively less frequently used and are mainly intracranial, subcutaneous, aerosol, and intratumoral injections [141]. Local administration of exosomes can increase their selective distribution to target organs or tissues whilst reducing systemic distribution and side effects [142]. Compared to healthy controls, transnasally administered MSC-Exos have exhibited significant accumulation in the brain under conditions of neurodegenerative changes and neurodevelopmental disorders, suggesting that in vivo pathological states may also influence the tissue distribution of exosomes [143].

Regarding tumor distribution specifically, exosomes tend to exhibit stronger enrichment at tumor sites while taking advantage of enhanced permeability and retention (EPR) (Fig. 7D) [144, 145]. The slightly acidic pH within the tumor microenvironment (TME) facilitates uptake. Compared to tumor cell-derived exosomes under normal culture conditions, tumor cell-derived exosomes treated with low pH and hypoxic conditions, similar to those in the TME, have stronger tumor-targeting specificity both in vivo and in vitro [146]. Exosomes from different cellular origins also show unique tissue selectivity in vivo. For example, B cell-derived exosomes are taken up by liver and spleen macrophages with high CD169 (sialic acid receptor) expression via surface-carried sialic acid [77], whilst tumor-derived exosomes exhibit homing phenomena, reflecting the targeted nature of exosome-delivered drugs [147].

## Exosomes in tumor therapy

### Delivering anti-cancer treatments

Exosomes are vesicles enclosed by a lipid membrane, with a size in the nanometer range. They possess the ability to evade phagocytosis by mononuclear macrophages, extend their presence in circulation, and have the capability to traverse through vascular walls and extracellular matrix. Additionally, they can even function as biological barriers. Low immunogenicity and high biocompatibility allow exosomes to be stable and widely distributed in vivo [12]. Owing to the rich biological properties of exosomes, the use of natural or engineered exosomes as drug delivery vehicles provides unique advantages and is expected to be a target delivery vehicle for nucleic acids, proteins, and chemical drugs. An ideal exosome carrier should have advantages such as a long circulation time, tumor site enrichment, deep tumor penetration, efficient intracellularization, and controlled drug release [148]. The application and in vivo characteristics of exosome vectors in different studies have been summarized in Table 2.

**Table 2:** Application of exosomes in tumor therapy

Application		Source	Cargo	Disease	Method of administration	Biodistribution and pharmacokinetics	Outcome/in vitro	Outcome/in vivo	Refs.
Drug delivery vectors	Small molecules	4T1 cell	DOX	Breast cancer	Intravenous injection	It was absorbed by the liver and spleen within 20 min of injection, and in addition to significant uptake in the liver and spleen, uptake was observed in the lungs and kidneys after 24 h	–	Significantly inhibited tumor growth	[107]
		MGC803 cell	DOX	Gastric cancer	Intravenous injection	The signal in the tumor area gradually increased within 48 h, whereas it decreased in the liver	Enhanced tumor cell killing effect	Significant tumor growth inhibition	[141]
		Bel7402 cell	DOX	Liver cancer	Intravenous injection	Increased tumor site accumulation and less normal organ accumulation were observed 24 h after injection compared with free drug	Exhibited the strongest cytotoxicity against tumor cells	Significant anticancer activity and prolonged survival time	[142]
		bone marrow MSCs	DOX	osteosarcoma	Intravenous injection	1 h after administration, exosome fluorescence was mainly observed in the liver region. At 12 h after administration, the fluorescence intensity of exosomes in the tumor area was strong	Significant cell uptake efficiency and antitumor effect	Tumor growth was significantly inhibited and cardiac toxicity was significantly reduced	[145, 146]
		RAW 264.7	paclitaxel	Lewis Lung Carcinoma	Intravenous injection	Exosomes were shown to colocalize with lung metastases 4 h after administration	Tumor cytotoxicity was more than doubled	Efficiently targeted lung metastases and significantly inhibited tumor growth	[144]
		Raw264.7	TRAIL + triptolide	Malignant melanoma	Intravenous injection	Its accumulation in the tumor site reached its peak at 6 h after injection and remained at the tumor site after 24 h	Inhibited proliferation, invasion, and migration and promoted apoptosis	Significantly inhibited tumor progression and reduced the toxicity of triptolide	[151]
	Biomacromolecules	YUSAC 2	Survivin-T34A + gemcitabine	pancreatic cancer	–	–	It significantly increased the apoptosis of tumor cells in a time-dependent manner	–	[158]
		HEK293T epithelial cells	CRISPR/Cas9 plasmid DNA	胰腺癌	Intravenous/intratumoral injection	–	Targeting mutant Kras G12D in pancreatic cancer cells and inducing target gene deletion	Inhibition of tumor growth in homologous subcutaneous and orthotopic models of pancreatic cancer	[164]
		Lewis lung carcinoma cell	miR-29a-3p	Lewis Lung Carcinoma	Intravenous injection	–	Tumor cell adhesion, colony formation, invasion, and proliferation were decreased	Targeted lung metastases and downregulated lung collagen	[167]
		HEK293T epithelial cells	iRGD modification Carnitine palmitoyltransferase 1A siRNA	Oxaliplatin resistant colon cancer	Intravenous injection	Targeting of the tumor approximately 6 h after injection	Inhibition of tumor cell proliferation and reversal of oxaliplatin resistance	Reversal of oxaliplatin resistance and inhibition of tumor growth	[171]
Immunotherapy		CAR-T cell	CAR	Human tumor cell lines expressing EGFR/HER2	Intravenous injection	-	Significant tumor cytotoxicity	Dose-dependent tumor growth inhibition	[185]
		human umbilical vein endothelial cell	Anti-PD-L1 + Anti-CD40 + cGAMP	Melanoma	Intravenous injection	At 2 h after injection, the tumor site showed obvious accumulation of exosomes, which continued to accumulate at the tumor site after 24 h	Tumor targeting and immune activation capabilities	Significantly delayed tumor growth and improved the survival rate of mice	[188]
Penetration of biological barrier		bEND.3 cell	DOX or paclitaxel	Brain cancer	Main vein injection	At 18 h after injection, exosomes were delivered across the BBB to the brain	Increased cytotoxicity in cancer cells	Xenograft tumor growth was significantly reduced	[192]
		bEND.3 cell	VEGF siRNA	Brain cancer	Main vein injection	Increased the distribution of siRNA in the brain by more than four times	Significant inhibition of VEGF	Xenograft tumors exhibited little fluorescence in the brain	[193]
		THP-1 induced M1 macrophages	Angiopep-2 + STAT3 siRNA	Glioblastoma	Intravenous injection	A strong accumulation of exosomes was observed in the brain 24 h after injection	Significant apoptosis of GBM cells	Significantly inhibited tumor growth and improved the median survival time of tumor-bearing mice	[194]
Improving the tumor microenvironment		HEK293T epithelial cells	STAT6-ASO	Colorectal cancer and hepatocellular carcinoma	Intravenous/intratumoral injection	95% of the intravenous dose was administered in the liver; After intratumoral administration, the highest mean fluorescence intensity was shown at TAM	Immunosuppressive M2 macrophages were reprogrammed into proinflammatory M1 macrophages	Potent antitumor activity and M1 macrophage reprogramming, as well as TME remodeling and CD8 + T-cell-dependent adaptive antitumor immune responses	[198]
		4T1 cancer cells	MnCO	metastatic breast cancer	Intravenous injection	High accumulation at the tumor site	Efficient killing and targeting ability of tumor cells	Significantly inhibited tumor growth and enhanced tumor radiosensitivity	[199]
		4T1 cancer cells	DOX-loaded long-circulating and pH-sensitive liposomes	breast cancer	Intravenous injection	–	Significantly reduced tumor cell viability	Stronger tumor killing effect and less acute toxicity as well as tissue/organ damage such as heart and spleen; reduction in the number of lung metastases	[201, 202]

DOX: doxorubicin; STAT: signal transducers and activators of transcription; ASO: antisense oligonucleotide; VEGF: vascular endothelial growth factor; MSCs: mesenchymal stem cells; TRAIL: tumor necrosis factor-related apoptosis-inducing ligand; T34A: Thr34 → Ala; siRNA: small-interfering RNA; CAR T-cells: genetically engineered T-cells expressing a chimeric antigen receptor; EGFR: epidermal growth factor receptor; HER2: human epidermal growth factor receptor-2; TAM: tumor-associated macrophages

#### Delivering small molecules

The targets of many antitumor chemotherapeutic drugs are intracellular; therefore, chemotherapeutic drugs must cross cell membranes before they can act. Chemotherapeutic drugs have difficulty in achieving the desired effect owing to their poor water solubility or short half-life. The lipid bilayer of exosomes protects the exosomally-loaded drug, which easily enters cells through the interaction of membrane proteins with the recipient cells. The greatest advantage of exosomes over synthetic drug carriers is their low immunogenicity and toxicity, which can reduce their clearance by the immune system and organ or tissue toxicity to a large extent [149].

The most serious adverse effects of Doxorubicin (DOX) are caused by its cardiotoxicity. Therefore, improving the target specificity of DOX to tumor tissues and reducing its concentration in cardiomyocytes are critical issues. Wei et al. [150] prepared BM-MSC-derived exosome-loaded Dox (Exo-Dox) by co-incubating Dox with BM-MSC-exosomes derived from bone marrow MSCs (BM-MSCs). Compared to free Dox, Exo-Dox exhibited significantly greater cellular uptake efficiency and antitumor effects in human osteosarcoma cells MG63, alongside decreased uptake efficiency and toxic effects in cardiomyocytes. This may have been related to the interaction between BM-MSC-derived exosomes and the surface membrane protein phase of human osteosarcoma cells. Further, in vivo results suggested that Exo-Dox could be used to target tumor sites and improve drug stability and tumor site accumulation. The number of Ki67-positive cells and cardiotoxicity were both significantly lower in the Exo-Dox group compared to the free Dox group [151].

Another factor limiting the therapeutic efficacy of chemotherapeutic agents is multi-drug resistance (MDR), which may originate from congenital or acquired acquisition. The presence of MDR reduces the tumor response rate to treatment and leads to the death of over 90% of patients with cancer receiving conventional chemotherapeutic agents or novel targeted agents [152]. Kim et al. [149] investigated different methods (room temperature incubation, electroporation, and mild sonication) to paclitaxel (PTX) loaded into macrophage exosomes and evaluated the feasibility of PTX-loaded exosomes (exoPTX) for the treatment of multi-drug resistant cancers. Of the three methods, sonication-treated exosomes showed high loading and sustained drug release, which may have been related to the incorporation of PTX during exosome membrane reconstitution. Compared to liposomes and polystyrene nanoparticles, exoPTX accumulated heavily in tumor cells in vitro and was more than onefold more cytotoxic to drug-resistant tumor cells. Similarly, in a mouse Lewis lung carcinoma pulmonary metastasis model, exoPTX showed near-complete co-localization and significant tumor growth inhibition out of tumor cells. Thus, exosomes as delivery vehicles facilitated in vivo targeting whilst also enhancing the antitumor effects of paclitaxel, suggesting that exosomes have great potential for delivering therapeutic agents in the treatment of drug-resistant cancers.

**Fig. 8:**
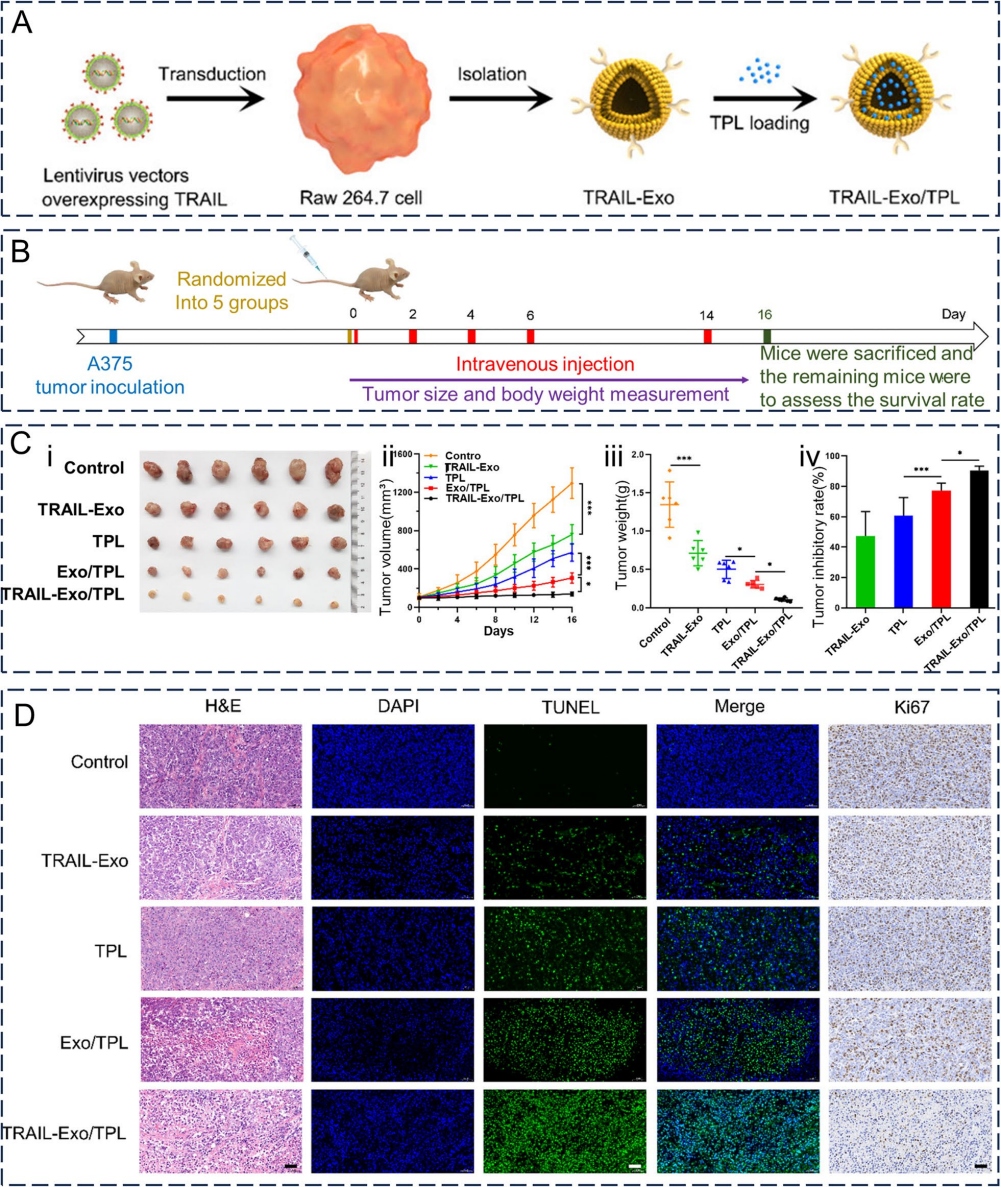
In vivo therapeutic effects of TRAIL-Exo/TPL. **A** Preparation of TRAIL—exobiology/TPL flow chart. **B** Flow chart of TRAIL-Exo/TPL in vivo experiments. **C** In vitro images of (i) tumors, (ii) tumor growth curves, (iii) tumor weights, and (iv) tumor inhibition rates of different treatment groups of mice after 16 days of intervention. **D** H&E, TUNEL and Ki67 staining of tumor sections from mice in different treatment groups after 16 days of intervention. (scale = 50 μm). Reproduced with permission. [156] Copyright 2021, American Chemical Society

The induction of apoptosis specifically in cancerous cells is one key function exhibited by Tumor necrosis factor (TNF)-related apoptosis-inducing ligand(TRAIL), which belongs to the TNF superfamily [153]. Specific highly expressed antigens such as death receptor 5 (DR5) are present on the surface of tumor cell membranes. Meanwhile, TRAIL is a high-affinity ligand for DR5 that transduces apoptotic signals by binding to DR5 [154]. However, TRAIL-based drugs such as recombinant human soluble TRAIL (rhTRAIL) have not yet achieved satisfactory therapeutic effects in clinical settings. This is because of the short half-life and insufficient targeting of rhTRAIL in vivo, which is limiting its clinical application [155]. Several recent preclinical studies have shown that TRAIL-bearing exosomes induce apoptosis whilst inhibiting cancer progression in vitro [156–158]. Jiang et al. [156] loaded exosomes secreted from TRAIL-overexpressing macrophages Raw264.7 to load triptolide (TP) with antitumor effects and subsequently obtained TP-loaded TP-based TRAIL-engineered exosomes (TRAIL-Exo/TP) (Fig. 8A). During in vitro assays, TRAIL-Exo/TP exhibited more significant melanoma growth inhibition and apoptosis promotion than free TP and TP-loaded exosomes alone. Notably, the exosomes themselves were virtually non-cytotoxic; however, the presence of TRAIL may have resulted in TRAIL-bearing exosomes displaying concentration-dependent cytotoxicity. In vivo experiments also showed that TRAIL-Exo/TP significantly inhibited tumor progression whilst reducing the toxicity of TPL in a melanoma nude mouse model, with good antitumor effects. The antitumor ability of TRAIL-Exo/TP was superior to that of TRAIL exosomes carrying TRAIL alone and loaded TP exosomes, suggesting a synergistic therapeutic effect of the drug combination delivery strategy (Fig. 8B–D). Furthermore, free TP exhibited hepatotoxicity, nephrotoxicity, and myelosuppression, whereas TRAIL-Exo/TP was biosafe and did not cause systemic toxicity or myelosuppression. In another study on the targeted delivery of TP, Gu et al. [159] successfully suppressed tumor growth in mice with tumors through loading TP onto arginine-glycine-aspartate (cRGD)-modified exosomes derived from human umbilical cord mesenchymal stromal cells(cRGD-Exo/TP), showing significant tumor targeting and a prolonged half-life of TP, thus ameliorating the short half-life and systemic toxicity of free TP.

#### Delivery of biomacromolecules

Unlike small-molecule drugs, proteins, peptides, and nucleic acid biomolecules are easily degraded and inactivated in vivo, whilst also facing a series of biological barriers such as cell membranes and endosomes in vivo, which limit the application of biomolecules in antitumor therapy. As carriers of intercellular information transfer, exosomes are naturally responsible for the delivery of bioinformatics molecules, have the inherent ability to cross biological barriers, and offer outstanding advantages for the delivery of biomolecular drugs. In addition, the co-delivery of small-molecule drugs with biomolecules using exosomes provides a new strategy for difficult-to-treat tumors [160].

**Fig. 9:**
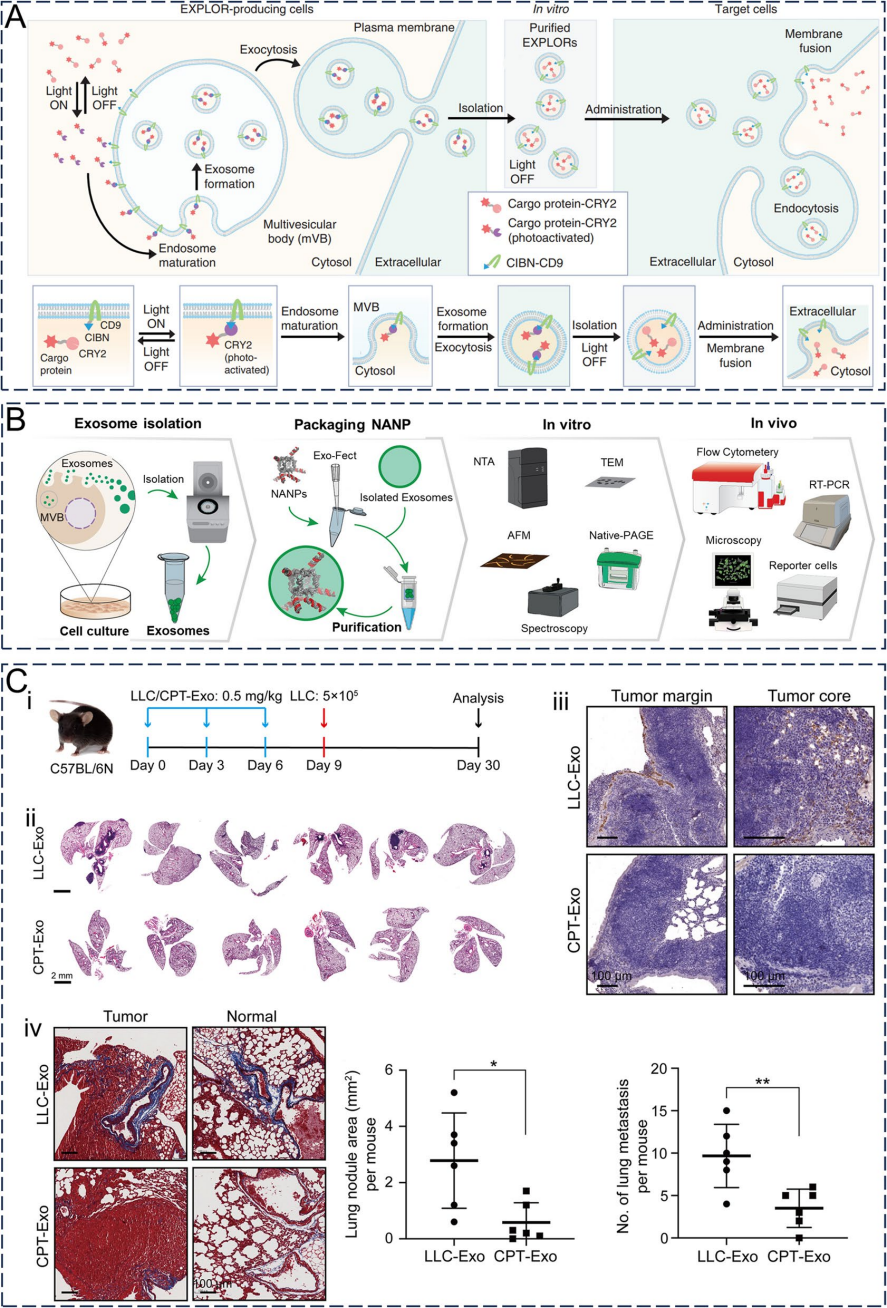
The application of exosomes delivering biological macromolecules in cancer therapy. **A** Schematic diagram of the synthesis and cell-targeted delivery of Exosomes for protein loading via optically reversible protein–protein interactions (EXPLORs). Reproduced with permission. [164] Copyright 2016, Springer Nature. **B** Synthesis and characterization of exosomes loaded with nucleic acid nanoparticles (NANPs). Reproduced with permission. [165] Copyright 2020, Elsevier Inc. **C** Schematic representation of cisplatin elicited exosomes loaded with miR-29a-3p inhibiting metastasis of Lewis lung carcinoma cells in mouse lungs (i) and HE staining image of the lung (ii), scale bar = 2 mm. (iii) By Masson staining showed two groups of mice lung tumors and the tumor area of total collagen protein expression level (scale = 100 microns). (iv) Collagen I expression levels (scale bar = 100 μm) at the tumor edge and tumor core in the lung of the two groups of mice by IHC staining (scale = 100 μm). Reproduced with permission. [172] Copyright 2022, Elsevier B.V

The inhibitor of apoptosis (IAP) survivin may be involved in the development of drug resistance whilst also serving as a potential prognostic marker for patients with pancreatic cancer. Inhibition of this protein expression significantly improves the sensitivity of tumor cells to chemotherapy or radiotherapy [161, 162]. Aspe et al. [163] previously inoculated engineered exosomes loaded with Survivin-T34A, which blocks survivin, into pancreatic adenocarcinoma cell lines and treated pancreatic cancer cells in combination with gemcitabine. Engineered exosomes loaded with Survivin-T34A were found to have significantly increased tumor cell apoptosis compared with free gemcitabine. In addition, the utilization of exosomes for protein loading via optically reversible protein–protein interactions (EXPLORs) has enhanced the effectiveness of exosome-mediated protein delivery and expedited their application as vehicles for delivering proteins in both tumor therapy research and oncology therapeutic investigations (Fig. 9A) [164].

Exosomes have shown great advantages in the delivery of nucleic acid drugs (Fig. 9B) [165]. Antisense oligonucleotides (ASOs) are an important type of deoxyribonucleic acid (DNA) that complement target mRNA and regulate RNA functioning through a variety of mechanisms. They are also highly selective therapeutic strategies for a variety of diseases associated with gene expression disorders [166]. Kamerkar et al. [167] showed that engineered exosomes loaded with the ASO of STAT6 or C/EBPβ (exoASO) reduced the expression of the corresponding target gene in primary human M2 macrophages in a dose-dependent manner. Through the reduced expression of target genes, exoASO leads to the reprogramming of M2 macrophages to M1 macrophages. In animal models, exoASO significantly inhibited tumor growth, whereas free ASO showed no significant effects on tumor growth. This suggested that exoASOs could target genes, induce effective reprogramming, and produce potent antitumor activity. Recently, CRISPR/Cas9-mediated genome editing has flourished, although common viral vectors (e.g., adenovirus) have limitations such as potential immunogenicity and insertional mutagenesis, whereas non-viral vectors (e.g., liposomes, polymers, and metal nanoparticles) have potential hazards such as low biocompatibility and organ toxicity [168]. These issues have limited the widespread use of CRISPR/Cas9 gene therapy. McAndrews et al. [169] previously showed that exosomes loaded with CRISPR/Cas9 plasmid DNA could target mutated KRAS G12D in pancreatic cancer cells as well as induce target gene deletion, thereby inhibiting tumor growth in pancreatic cancer homologous subcutaneous and in situ models. This suggests that exosomes are promising alternative delivery vehicles for CRISPR/Cas9 gene therapy.

In addition to DNA, exosomes can deliver ribonucleic acids (RNA). MicroRNA (miRNA) is non-coding RNA that regulates gene expression by binding to mRNA [170]. The regulation of tumor-associated gene expression by therapeutic miRNAs is emerging as a promising strategy for tumor therapy [171]. Yan et al. [172] found that cisplatin promoted the accumulation of miR-29a-3p-loaded lung tumor cell-derived exosomes in tumor cells and induced an approximately 30-fold upregulation of circulating exosomal miR-29a-3p in vivo. Furthermore, exosomal miR-29a-3p exhibited the ability to target and downregulate collagen in the lung, which could provide a pro-metastatic microenvironment for tumor cells, suggesting that exosomal miR-29a-3p has the potential for treating lung tumors (Fig. 9C). They also used liposomes to mimic exosomes for loading miR-29a-3p and observed similar in vivo effects as exosomal miR-29a-3p. Alternatively, miRNA-based therapies can interfere with the expression of target miRNAs in tumor cells by delivering inhibitors of specific miRNAs or anti-miRNA oligonucleotides (AMOs) [173]. siRNAs are short-sequence double-stranded RNAs that complement target mRNAs, leading to gene silencing [174]. When siRNA is used in tumor therapy, it is unstable and highly susceptible to degradation [175]; therefore, using exosomes to deliver siRNA could present a promising approach. Lin et al. [176] used iRGD-modified exosomes loaded with a carnitine palmitoyltransferase 1A (CPT1A) siRNA (siCPT1A). CPT1A is a key enzyme for fatty acid oxidation (FAO), which may play an important role in oxaliplatin resistance in colon cancer. Compared to natural exosomes, iRGD-modified exosomes showed efficient targeting of colon cancer cells in vivo. Furthermore, loading with siCPT1A also allows for targeted delivery of siCPT1A to tumors to inhibit FAO, thus effectively overcoming the resistance to oxaliplatin and suppressing the proliferation of tumors. Furthermore, exosomes have the capability to transport circRNAs, long noncoding RNA (lncRNA), short hairpin RNA (shRNA), and aptamers, thus providing powerful vectors and tools for gene therapy and therapeutic nucleic acid delivery [166].

### Application of immunotherapy

**Fig. 10:**
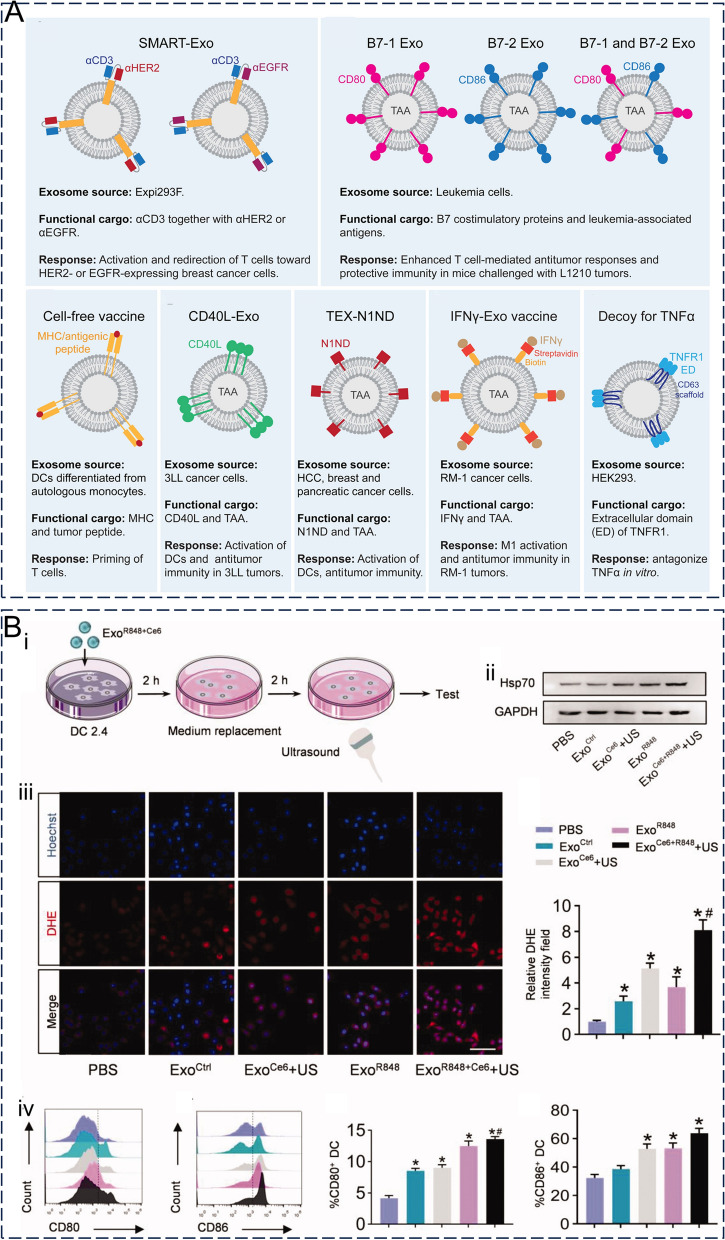
Strategies and applications of exosome-enhanced immunotherapy. **A** Engineered exosomes for cancer immunotherapy. Reproduced with permission. [178] Copyright 2020, Wiley. **B** HEK293-derived exosomes loaded with R848 (immunoadjuvant) and Ce6 (sonosensitizer) promoted DC2.4 maturation. (I) ExoR848 + Ce6 intervention DC2.4 schematic diagram. (ii) Western blot analysis of Hsp70 in DC2.4 from different treatment groups. (iii) The production of ROS in the different treatment group DC2.4 (scale = 100 microns) and statistical analysis. (iv) Flow cytometry and statistical analysis of CD80 and CD86 expression in DC2.4 of different treatment groups. Reproduced with permission. [140] Copyright 2022, Informa UK Limited

Tumor immunotherapy exerts an effective antitumor response by reactivating the patient's immune system, in particular, CD8 + T cells. Exosomes are used for immunotherapy in two ways: The activation of the immune response mediated by exosomes to stimulate antigen presentation and the killing activity of autoimmune cells in the intrinsic and adaptive immune systems to inhibit tumor growth, that is, cancer vaccines. Cancer vaccines primarily include peptide, cellular, viral, and genetic vaccines (Fig. 10A) [177, 178].

Releasing exosomes from antigen-presenting cells (such as B cells or DC) has the potential to initiate and maintain a robust immune response against tumors and thus are mostly cellular vaccines belonging to DCs [179]. DEX performs better than DCs as a delivery vehicle for tumor antigens since it is more stable and targeted, in addition to its immunostimulatory function [180, 181]. Wang et al. [182] developed an ovalbumin (OVA)-specific, DC (DCOVA)-DC-released, exosome (EXO)-targeted, T cell-based (OVA-Texo) system. OVA-Texo activated mT cells by directly stimulating antigen-specific CD8 + cytotoxic T lymphocytes (CTL) and activating the mTORC4 pathway to convert depleted T cells into CTL to stimulate a larger CTL antitumor response. Additionally, both tumor- and normal cell-derived exosomes have the ability to stimulate antitumor immune responses by functioning as vesicles that present tumor-associated antigens (TAA) to DCs. These TAA-carrying TEX are efficiently taken up by DCs, enabling the effective presentation of antigens on MHC molecules to corresponding T cells [183, 184]. Considering that TEX originate from a tumor cell, it has characteristics similar to those of tumor cells. Although TEX exhibits tumor-immunostimulatory effects, it also has immunosuppressive and tumor-promoting potential [185]. For example, pancreatic ductal adenocarcinoma-derived (PDAC)-derived exosomes inhibit complement-mediated cytotoxicity against tumor cells [186]. Therefore, the potential of TEX to promote immunosuppression and immune evasion must be considered before it can be used in cancer vaccines.

Chimeric antigen receptor (CAR)-T cells represent an innovative and promising approach to tumor immunotherapy, particularly for the treatment of B-cell lymphoma and acute lymphoblastic leukemia. The principle of this approach is the recognition of specific antigens on the surface of tumor cell membranes by the extracellular structural domains of genetically engineered T cells expressing recombinant receptors. This in turn triggers the activation of T cell receptor (TCR) signaling by intracellular structural domains, leading to antigen-targeted cytotoxicity as well as target cell death [187]. However, Cytokine release syndrome (CRS), immune effector cell-associated neurotoxicity syndrome (ICANS), and hematotoxicity (including cytopenia, immune reconstitution dysfunction, and hypogammaglobulinemia) and “on-target, off-tumor” response, as well as barriers to the immunosuppressive tumor microenvironment. The negative effects on CAR-T cell activity and persistence pose a challenge for CAR-T cell therapy. For these reasons, CAR-T cell therapy has limited therapeutic efficacy in the clinical application of solid tumors compared to hematologic malignancies [188, 189]. Recent studies have shown that CAR-T cell-derived exosomes as a cell-free therapy not only retain most of the functions of CAR-T cells but also have greater therapeutic efficiency, higher controllability, lower toxicity, and safety, and could even replace CAR-T cells as a powerful tool for solid tumor treatment. In addition, since CAR-T cell-derived exosomes do not express programmed cell death protein 1 (PD1) on their surface, PD-L1 on the surface of tumor cells cannot inhibit exosomes in the same way as the antitumor immunity of CAR-T cells [190].

The other is the delivery of immunotherapeutic agents such as immune checkpoint inhibitors (Fig. 10B) [140], where monoclonal antibodies targeting specific molecules can be expressed on the surface of exosomes. Immune checkpoint blockade (ICB) activates antitumor cytotoxic T-cell responses and improves the efficiency of cancer therapy by blocking regulatory receptors expressed on immune or tumor cells, with PD-1/PD-L1 and CTLA-4 being the key targets for immune checkpoint blockade therapy [191]. Despite the commercialization of anti-CTLA-4 and anti-PD-L1 monoclonal antibodies, they still have drawbacks such as high off-target effects, low objective response rates, and potential immune-related side effects [192]. To address these issues, Fan et al. [193] engineered exosomes by modifying anti-PD-L1 and anti-CD40 on the surface of human umbilical vein endothelial cells (HUVECs) before loading them with cGAMP, a dual-targeting exosome-loaded drug (cGAMP@dual-anti-Exos). cGAMP@dual-anti-Exos accumulates at the tumor site, with anti-PD-L1 then binding to PD-L1 on the tumor cell surface and blocking immune checkpoint molecules. The engineered exosomes were then used to stimulate DCs twice with anti-CD40 and cGAMP. By combining immunotherapy with two antibodies and one drug, both tumor suppression and the immune response were enhanced, whereas immune escape was inhibited, providing a relatively safe tool for combination immunotherapy.

### Penetration of biological barrier

**Fig. 11:**
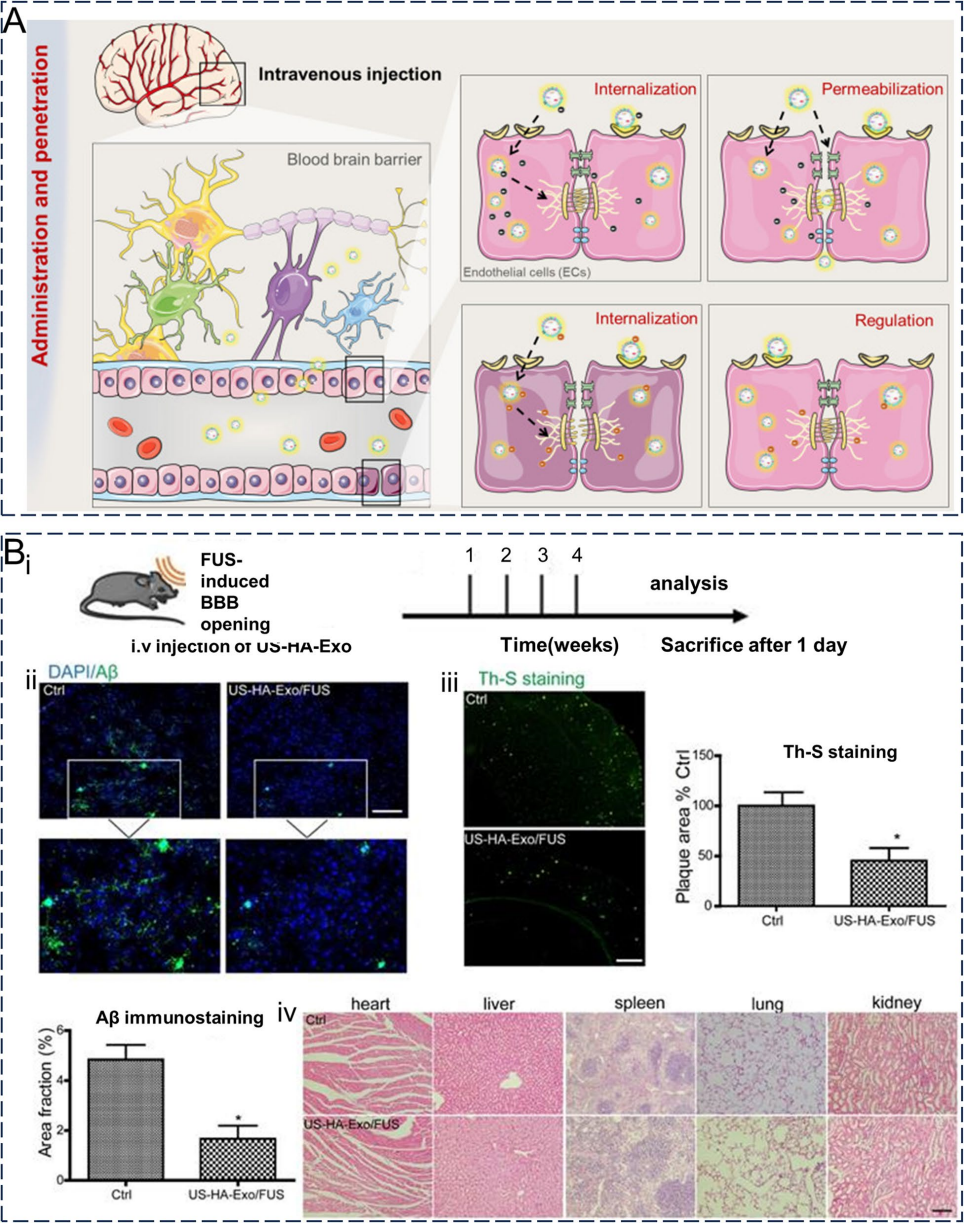
Exosomes carry therapeutic agents through biological barriers to exert therapeutic effects. **A** Schematic diagram of the mechanism of exosomes penetrating the blood–brain barrier. Reproduced with permission. [197] Copyright 2021, Ivyspring International Publisher. **B** FUS-BBB opening facilitates exosome delivery to the brain. (i) Schematic diagram of the experimental procedure. (ii) Aβ immunostaining images of 10-month-old APP/PS1 mice (scale bar = 100 μm) and percentage area of quantitative positive amyloid β staining. (iii) Thioflavin-S staining (scale scale = 100 μm) and quantification of Aβ plaques in brain. (iv) HE staining of vital organs in mice (scale bar = 100 μm). Reproduced with permission. [198] Copyright 2021, Elsevier B.V

The blood–brain barrier (BBB) is the interface between blood flow and brain parenchyma and acts as an anatomical and physiological barrier to prevent harmful substances from entering the brain or gastrointestinal tract. It also serves as an existing barrier to antitumor drugs, thus limiting the effective delivery of most therapeutic agents to the tumor. This restricted passage of antitumor drugs through the BBB is mainly attributed to active efflux transporters (AETs) [194]. P-glycoprotein (P-gp) and breast cancer resistance protein (BCRP) are the two most common AETs that are associated with drug resistance in multiple tumors and limit the brain penetration of antineoplastic drugs [195]. More than half of the commercially available antitumor drugs are recognized by P-gp, which prevents drug entry into the brain parenchyma via the transcytosis pathway. Although a partial blood–brain tumor barrier (BBTB) with increased permeability due to abnormal blood vessel formation is present in brain tumors, the BBTB in most brain tumor areas is closer to the intact BBB [196]. To address the issue of poor drug accessibility in brain tumors, in addition to developing new therapeutic strategies for overcoming BBB/BBTB, the selection of suitable drug delivery vehicles presents a promising approach. Exosomes are rich in membrane proteins and specific lipids that confer their ability to cross biological barriers and have been shown to cross the blood–brain barrier and gastrointestinal tract for the brain or gastrointestinal delivery of biomolecules or drugs (Fig. 11A, B) [197, 198]. The targeting ability of unmodified exosomes is not strong, however, with only approximately 0.5% reaching the brain [29]. However, they have been able to significantly improve the effective concentration and therapeutic efficacy of antitumor drugs at the tumor site [199, 200]. Surface modification can also further improve the targeting ability of exosomes. Glioblastoma multiforme (GBM) is one of the most difficult and aggressive tumors to treat, with the difficulty of accessing therapeutic agents in the tumor region being one of the primary reasons for poor prognosis and inevitable recurrence. Liang et al. [201] also prepared exosomes loaded with angiopep-2 (An2)-functionalized signal transducers and activators of transcription 3 (STAT3)-siRNA to target GBM cells and BBB endothelial cells through An2 binding to low-density lipoprotein receptor-related protein 1 (LRP-1), to promote Exo-An2-siRNA penetration into the BBB and target GBM. STAT3 is one of the GBM therapeutic targets, whilst Exo-An2-siRNA is taken up by GBM cells and releases siRNA to silence STAT3, significantly inhibiting downstream target oncogene transcription, and ultimately leading to significant apoptosis in GBM cells. In vivo, Exo-An2-siRNA showed the strongest inhibition of tumor growth (compared to Exo-An2, free siRNA, and PBS) alongside outstanding improvement in the median survival time of hormone-treated mice, whilst also exhibiting excellent accumulation and tumor-homing properties.

### Improvement of the tumor microenvironment

**Fig. 12:**
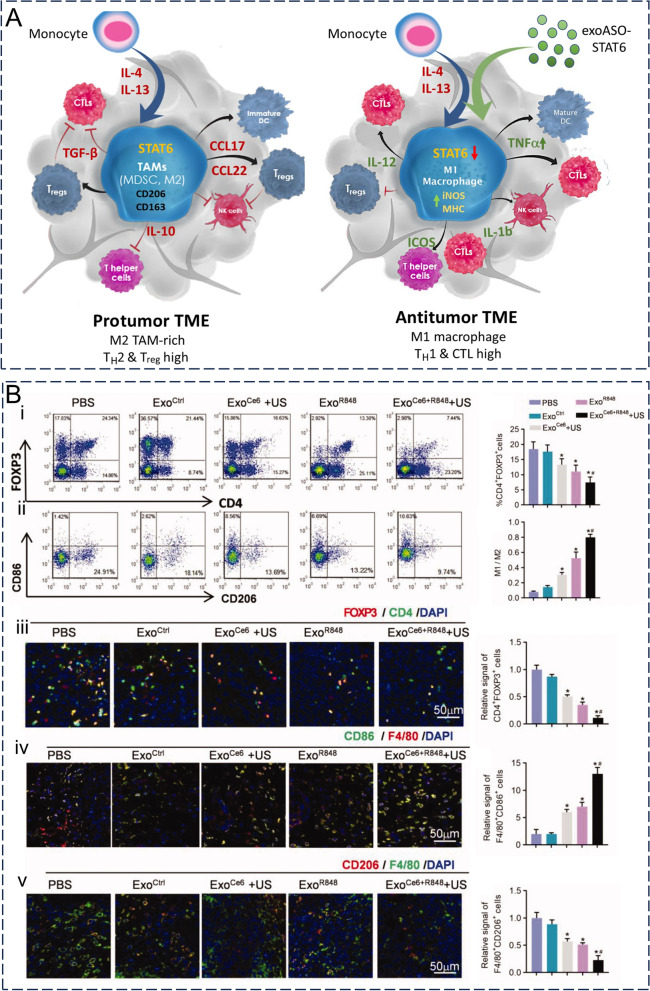
Exosomes act as vectors to regulate the tumor microenvironment. **A** Exosomes loaded with STAT6-targeted ASO reprogrammed M2 tumor-associated macrophages (TAMs) from tumor-promoting M2 to anti-tumor M1 by knocking down STAT6. Reproduced with permission. [205] Copyright 2022, Kamerkar, Leng, Burenkova, et al. **B** ExoCe6 + R848 improves the immunosuppressive tumor microenvironment. (i) Flow cytometry images and proportions of Treg cells (CD4 + FOXP3 +) in different treatment groups. (ii) Flow cytometry images and proportions of M1 (CD86) and M2 (CD206) in different treatment groups. (iii) Immunofluorescence staining and statistical results of Foxp3 and CD4 in tumor sections of different treatment groups. (iv) Immunofluorescence staining and statistical results of CD86 and F4/80 in tumor sections of different treatment groups. (v) Immunofluorescence staining and statistical results of CD206 and F4/80 in tumor sections of different treatment groups. Scale = 50 μm. Reproduced with permission. [140] Copyright 2022, Informa UK Limited

The TME is composed of cellular and noncellular components. Cellular components include fibroblasts, endothelial cells, and immune cells (e.g., macrophages, T and B lymphocytes, and DCs), whilst non-cellular components include soluble factors (e.g., cytokines, growth factors, and chemokines), extracellular matrix proteins (laminin, fibronectin, and collagen), and EV [202]. Macrophages have two phenotypes, M1 and M2. M2-type tumor-associated macrophages (TAMs) in the tumor microenvironment are usually considered the major subtype of tumor immunosuppression and may reduce the effectiveness of immunotherapy [203]. Reprogramming TAMs from the M2 to the M1 subtype is one feasible approach to reversing tumor immunosuppression (Fig. 12B) [140]. Controlling the M2 phenotype of TAMs under pathological conditions involves key transcription factors, including signal transducer and activator of transcription 6 (STAT6) [204]. Engineered exosomes (exoASO-STAT6) modified with the antisense oligonucleotide (ASO) of STAT6 preferentially target M2 macrophages. Moreover, exoASO-STAT6 delivered ASO over tenfold more efficiently than free ASO. Additionally, exoASO-STAT6 effectively reprogrammed immunosuppressive M2-type macrophages into pro-inflammatory M1-type macrophages in vitro, which may have been dependent on the suppression of STAT6 pathway gene expression in M2-type macrophages and the expression of M1-type macrophage gene markers. Similar to the in vitro results, ExoASO-STAT6 also produced effective M1 macrophage reprogramming in mouse models of colorectal and hepatocellular carcinoma. This led to TME remodeling and a CD8 + T cell-dependent adaptive antitumor immune response. Furthermore, ExoASO-STAT6 showed potent antitumor activity in both mouse tumor models (> 90% inhibition of tumor growth), whereas free ASO at equivalent drug doses did not exhibit any therapeutic effects (Fig. 12A) [205].

The effectiveness of exosome-based therapies is limited by factors such as angiogenesis, fibrotic signaling, hypoxia in the TME, which can hinder drug delivery and intra-tumor infiltration whilst increasing tumor resistance to drugs [202]. Therefore, methods of exerting effective therapeutic efficiency in the tumor microenvironment are important to investigate for tumor drug therapy. Zhu et al. [206] previously developed tumor-derived exosome-hybrid nanovesicles (MMV) loaded with the mitochondrial toxicity drug MnCO, which relies on the targeting ability of exosomes to exhibit tumor-selective accumulation in vivo. Tumor cells overproduce H2O2, whilst MnCO then reacts with H2O2 to produce CO, which subsequently damages the mitochondria, thereby killing tumor cells. Considering that normal cells do not produce excessive H2O2, tumor cells are selectively killed. This also prevents leakage and has a low CO loading rate during delivery. Compared to the neutral environment under physiological conditions, the tumor microenvironment usually appears acidic, which may be related to the high rate of glycolysis alongside increased lactate production [207]. This property, which is different from that of normal tissues, provides new ideas for potential antitumor drug delivery. Gomes et al. [208] fused breast cancer cell-derived exosomes with DOX-loaded long-circulating and pH-sensitive liposomes (SpHL-DOX) to obtain the hybrid exosome ExoSpHL-DOX. The results subsequently showed that ExoSpHL-DOX was sensitive to environmental pH and could selectively release antitumor drugs in an acidic environment. ExoSpHL-DOX released 96.6 ± 0.2% of DOX in pH 5.0 medium compared to 70.1 ± 1.7% in pH 7.4 medium. Compared to free DOX, ExoSpHL-DOX also induced stronger tumor-killing effects with less acute toxicity and tissue/organ damage in the heart and spleen in vivo, whilst the blood concentration of DOX was also further enhanced. In addition, ExoSpHL-DOX was therapeutically effective against lung metastases from breast cancer, reducing the number of lung metastases [209].

### Liquid Biopsy

Liquid biopsies are usually based on circulating tumor cells (CTCs), circulating tumor DNA (ctDNA), and exosomes derived from tumor sites as markers, which often show abnormalities in the early stages of tumor development before imaging changes and traditional tumor markers. They can also be used in healthy and high-risk populations for early warning and screening, treatment effect monitoring, and prognosis assessment, as well as tumor MRD and recurrence monitoring in healthy and high-risk populations [210]. Compared to traditional tissue biopsies, liquid biopsies have a reduced risk, cost, and detection time, whilst also being non-invasive [211].

Tumor-derived exosomes have potential applications in tumor diagnosis, prognosis, monitoring, and metastasis since they carry tumor-specific information, mediate communication between tumor cells, participate in the regulation of the tumor microenvironment, and promote tumor growth and metastasis [11]. Li et al. [212] designed and implemented a multicenter prospective study in a cohort of patients with esophageal squamous cell carcinoma (ESCC) and healthy volunteers, including a pilot cohort for RNA sequencing (three ESCC patients and three controls each) and a discovery cohort for further validation (33 ESCC patients and controls each). RNA sequencing of the salivary exosomes was performed to identify tRNA-derived small RNA (tsRNA). It was found that a double marker consisting of tRNA-GlyGCC-5 and a previously unidentified small RNA (named sRESE because of its origin in the saliva exosomes of ESCC patients) could distinguish ESCC patients from controls with high sensitivity (90.50%) and specificity (94.20%). These findings further demonstrated the feasibility and application of exosomes as biomarkers for tumor diagnosis. Furthermore, Nakano et al. [213] performed a microarray analysis of serum exosomes in patients with hepatocellular carcinoma (HCC) after living donor liver transplantation (LDLT). Microarray analysis was performed and miR-92b was found to have been highly expressed in these exosomes. In addition to the high expression of miR-92b in the serum exosomes of pre-LDLT HCC patients, high levels of miR-92b were also maintained in patients with recurrent HCC after LDLT. Therefore, they evaluated the predictive value of miR-92b expression for early hepatocellular carcinoma recurrence after LDLT. The results showed that the predictive accuracy of pre-LDLT miR-92b expression for early HCC recurrence after LDLT was high (sensitivity and specificity: 71.4% and 62.8%, respectively). If a combination of alpha-fetoprotein (AFP) and miR-92b was used for prediction, the accuracy of early hepatocellular carcinoma recurrence prediction after LDLT could be further improved.

## Conclusions and prospects

Current tumor treatment strategies, such as chemotherapy, gene therapy, and immunotherapy, may have difficulties in achieving the expected efficacy during clinical application owing to limitations such as weak targeting, low effective concentration, and local or systemic toxicity. Improving the efficiency of drug or gene delivery, starting with the selection of appropriate carrier types, is one of the main options for improving the effectiveness of oncological treatments. Exosomes, as delivery carriers, combine the advantages of cell-based drug delivery and nanomaterials, with their superior biocompatibility and nanosize enabling effective drug delivery. Compared with cell-based drug delivery, exosomes not only inherit most of the functions of source cells but are also easier to control and store and are safer. In addition, compared with nanomaterial-based drug delivery systems, the lipid bilayer, surface membrane proteins, and nanosized structure of exosomes ultimately enable them to overcome various biological barriers, low immunogenicity, natural targeting, and stability for long-distance delivery in vivo [145]. An increasing number of studies have shown that exosomes are promising vehicles and tools for delivering oncological therapeutics. The comparative advantages and disadvantages of exosomes compared with other similar carriers are summarized in Table 3.

**Table 3:** Comparison of exosomes with other delivery vectors

Type of drug delivery vectors	Advantage	Disadvantage
Exosome	Good biocompatibility Stability Low immunogenicity and cytotoxicity Targeting ability Crossing of biological barriers	Lack of standardized separation and purification methods Heterogeneity Low drug loading efficiency Limited mass production
Liposome	Good biocompatibility Easy surface modification Wide adaptability to loaded drugs Long blood circulation time High bioavailability and safety Similar to cell membrane structure	Long-term application only for small molecule drug delivery Low drug loading rate Poor stability, easy oxidation of phospholipids, susceptible to metals, radiation, high temperature, PH, and enzymes Induces a toxic immune response in vivo, mainly in the liver
Polymer nanoparticle	Good biocompatibility, biodegradability High therapeutic drug load Easy absorption, controlled drug release Ligand or targeted modification of polymer surface can achieve multifunctional drug delivery	Easy to bind to negatively charged nonspecific cells or proteins High cytotoxicity Low gene transfection efficiency
Micelle	Enter living cells without the use of transfection agents Long retention time in vivo Good tissue permeability、 Biocompatibility, biodegradability Easy structure modification and special "core–shell" structure Uniformity, small volume	Poor physical stability Easy to cause drug leakage and sudden release
Inorganic/metallic nanoparticles	Small and uniform size Unique physical and chemical properties, such as optical, magnetic, electrical, acoustic Easy to degrade in a short time Nonimmunogenic	Low biocompatibility Biotoxicity

Reproduced with permission. [214]. Copyright 2023, Xinyu Lin, Ying Wang, Kai Fang, et al

Although exosomes have achieved outstanding results in recent years as drug carriers for the targeted therapy of tumors, there are still many shortcomings limiting their clinical application. The main issues are as follows: (1) The in vivo function and safety of exosomes remain controversial. Owing to their biological activity, the safety of exosomes should be considered when used as delivery vehicles. For example, tumor cell-derived exosomes contain tumor-supporting components that promote tumor growth, invasion, and metastasis, thus there are risks associated with inducing immunosuppression or accelerating tumor progression. Therefore, more clinical studies on exosome therapy are needed to focus on the safety and toxicological characteristics of human experiments. (2) The state of the source cells, modifications upstream and downstream, have an impact on the composition and characteristics of exosomes, resulting in diverse therapeutic outcomes. Therefore, controlling the stability of the source cell culture environment and developing gentle and effective exosome processing techniques could help reduce variation between batches whilst maintaining stable exosome structure and properties [215]. (3) Existing isolation techniques can only produce small amounts of exosomes, which are costly to produce on a large scale [216]. Factors such as stimulation of cells to secrete more exosomes (e.g., regulation of intracellular calcium levels, external stress, cytoskeletal blockade, drug effects, and gene expression [49]), development of more efficient isolation and purification methods (e.g., tangential flow filtration [217], asymmetrical flow field-flow fractionation [218]) or exploring new sources of exosomes (e.g., milk [219], bacteria [220], plants [221], etc.) could help to address this problem. (4) There is a lack of comprehensive understanding regarding selective cellular uptake mechanisms for exosomes along with their intracellular distribution patterns leading to possible off-target phenomena. (5) The risk of altering the orientation of membrane proteins when performing membrane-disrupting manipulations such as drug delivery to exosomes, which may result in recognition by the immune system and subsequent adverse reactions. Recently, in situ exosome-based drug delivery strategies based on organism generation have provided researchers with a new idea, which involves transforming in vitro isolation, drug loading, and modified delivery into in situ release at the lesion site, thus avoiding the risk of altering exosome properties and characteristics [222]. (6) Criteria for manufacturing, loading efficiency, purification, storage, use, stability duration, dose, and application are yet to be determined [223]. (7) There are significant differences between laboratory studies and clinical industrialization studies on exosome drug delivery systems, with only a few exosome-based therapies entering clinical trials. Therefore, research on exosomes as drug carriers should focus more on in vivo safety and toxicological profiles, expansion of exosome sources, gentler and more efficient isolation, drug delivery, optimization of modification techniques, standardization from production to application, and detailed mechanisms of selective cellular uptake and intracellular distribution. This could help to bridge the gap between laboratory studies and clinical translation. In conclusion, exosome research remains in its early stages, although exosome delivery systems have shown promising applications in oncology therapy. The design of appropriate exosome delivery systems could maximize therapeutic efficacy whilst effectively facilitating the clinical translation of exosomes for oncology therapeutic applications.

## Data Availability

Not applicable.
